# Trivalent Gd-DOTA reagents for modification of proteins†
†Electronic supplementary information (ESI) available: Synthetic details for known compounds; materials and methods for bioconjugation reactions; copies of spectra of new compounds and compounds prepared according to new procedures. See DOI: 10.1039/c5ra20359g



**DOI:** 10.1039/c5ra20359g

**Published:** 2015-11-11

**Authors:** Martin J. Fisher, Daniel J. Williamson, George M. Burslem, Jeffrey P. Plante, Iain W. Manfield, Christian Tiede, James R. Ault, Peter G. Stockley, Sven Plein, Azhar Maqbool, Darren C. Tomlinson, Richard Foster, Stuart L. Warriner, Robin S. Bon

**Affiliations:** a School of Chemistry , University of Leeds , LS2 9JT , UK . Email: r.bon@leeds.ac.uk; b Astbury Centre for Structural Molecular Biology , University of Leeds , UK; c School of Molecular and Cellular Biology , University of Leeds , UK; d Leeds Institute of Cardiovascular and Metabolic Medicine , University of Leeds , UK; e Multidisciplinary Cardiovascular Research Centre , University of Leeds , UK

## Abstract

The development of novel protein-targeted MRI contrast agents crucially depends on the ability to derivatise suitable targeting moieties with a high payload of relaxation enhancer (*e.g.*, gadolinium(iii) complexes such as Gd-DOTA), without losing affinity for the target proteins. Here, we report robust synthetic procedures for the preparation of trivalent Gd-DOTA reagents with various chemical handles for site-specific modification of biomolecules. The reagents were shown to successfully label proteins through isothiocyanate ligation or through site-specific thiol–maleimide ligation and strain-promoted azide–alkyne cycloaddition.

## Abstract 2

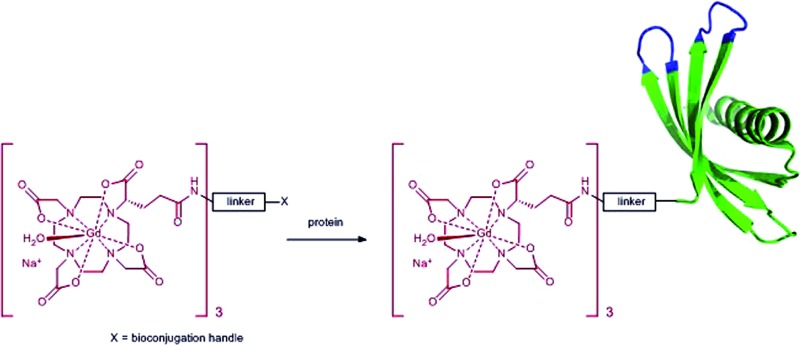
The development of novel protein-targeted MRI contrast agents crucially depends on the ability to derivatise suitable targeting moieties with a high payload of relaxation enhancer without losing affinity for the target proteins.

## Introduction

Magnetic resonance imaging (MRI) is widely used for non-invasive imaging of physiological processes in both clinical medicine and pre-clinical research. MRI is especially advantageous because, in comparison to other imaging modalities, it offers the opportunity to image deep into tissue without the need for ionising radiation, and because it provides 3D images with sub-millimeter spatial resolution.^1–3^ Most MRI methods rely on ^1^H NMR signals of water protons, so signals depend on their concentrations and relaxation times (*T*_1_ and *T*_2_). Chemical contrast agents, for example those based on gadolinium(iii) or manganese(ii) complexes or on iron oxide nanoparticles, can significantly enhance the sensitivity of MRI, and have already had major impact on (pre)clinical imaging. All clinically approved MRI contrast agents that are currently in use are based on the paramagnetic Gd^3+^ ion,^4^ which can shorten the spin–lattice relaxation time (*T*_1_) of water in its coordination spheres. Efficient Gd^3+^-based MRI contrast agents undergo rapid exchange of inner-sphere water molecules with bulk water, resulting in significant enhancement of MR contrast by low micromolar concentrations of Gd^3+^.^1–3^

Targeted MRI contrast agents consist of a targeting moiety and one or more gadolinium complexes. The targeting moiety is usually a small molecule or peptide. These reagents can be used to localise specific proteins – typically proteins present in the blood or extracellular matrix, or extracellular domains of membrane proteins – *in vivo* with high spatial resolution. Examples are MRI contrast agents targeted to serum albumin, collagen, fibrin,^2,3^ and various abundant receptors (*e.g.*, progesterone, folate, dopamine glutamate).^1^ In addition, a contrast agent based on CTB (cholera toxin subunit B) has been used to successfully image neuronal connections *in vivo*.^5^

The development of protein-targeted MRI contrast agents crucially depends on the availability of suitable protein-targeting moieties that can be labelled efficiently with (multiple) gadolinium complexes while preserving: (1) affinity of the targeting moiety for its protein target; (2) strong binding of toxic Gd^3+^ to its chelating ligand; (3) relaxivity of the gadolinium complex; (4) rapid tissue penetration/target binding and clearance of unbound reagents; and (5) clearance of all reagent from the body before metabolism-related release of Gd^3+^.

For many proteins, no suitable small molecule ligands are available, and the use of biomolecules as targeting moieties would be desirable. For example, antibody- and affibody-based MRI contrast agents have been developed that enable the targeted imaging of EGFR and Her-2,^6^ including a two-component approach based on a biotinylated antibody and Gd-DTPA-labelled avidin to increase the gadolinium payload (with a detection limit of *ca.* 10^6^ receptors per cell).^7^ Affibodies and other antibody mimetics have significant advantages over antibodies as targeting moieties: they are smaller, causing rapid tissue penetration and blood clearance; they can be selected rapidly *in vitro* against a wide range of biomolecules; they are easier to produce in homogeneous batches that are devoid of post-translational modifications; and they can be labelled site-specifically with suitable chemicals.

**Fig. 1:**
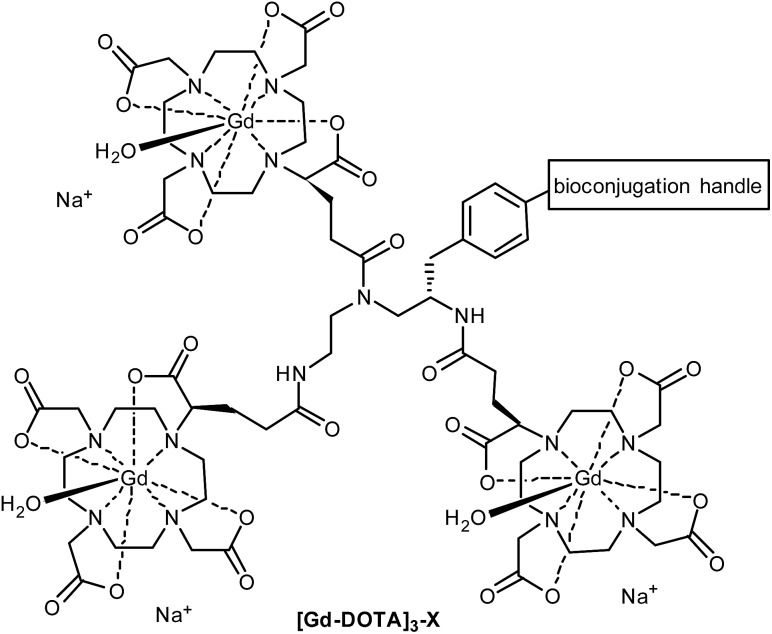
Generic structure of trivalent Gd-DOTA reagents **[Gd-DOTA]_3_-X** described in this study (X = reactive handle for chemoselective conjugation to biomolecules).

Macrocyclic ligands such as DOTA and DO3A are metabolically more stable than linear ones such as DTPA, and they bind Gd^3+^ much more tightly (p*K*_D_ = 28 for DOTA *vs.* 22 for DTPA),^8^ minimising potential toxicity issues. DOTA can be linked to targeting moieties through amide bond formation with one (or more) of its carboxylic acids. However, the change of a coordinating carboxylate to an amide slightly reduces the binding constant of the Gd-DOTA complex. More importantly, this substitution significantly slows the water exchange rate of the complex (a limiting factor when imaging slow-tumbling molecules).^1,3^ Our aim was to generate a toolbox of trivalent Gd-DOTA reagents that already contain Gd^3+^ and can be used to selectively label any targeting moiety (*i.e.* small molecules, peptides and biomolecules) in one simple step. The trivalent Gd-DOTA reagents were based on DOTA-GA in combination with a diethylenetriamine linker (Fig. 1). DOTA-GA contains a glutamic acid side chain that allows chemical modification without negatively affecting the water exchange rate of the Gd-DOTA complex. In addition, the limited rotational freedom of the short triamine linker would ensure minimal loss of relaxivity through rotational motion (a limiting factor when using clinically prevalent mid-range scanners).^1,3^

One trivalent complex **[Gd-DOTA]_3_-X** (Fig. 1, X = NCS; compound **14**) has previously appeared in the patent literature.^9^ However, DOTA chemistry is notoriously sensitive to impurities and, in our experience, the details disclosed in this patent are insufficient to allow straight-forward preparation of trivalent Gd-DOTA reagents. Here, we provide detailed (optimised) procedures for the synthesis and purification of one known and two new trivalent Gd-DOTA reagents **[Gd-DOTA]_3_-X**. In addition, we report on bioconjugation studies for the modification of proteins such as antibody mimetics.

## Results and discussion

### Synthesis of **(*R*)-*^t^*Bu_4_-DOTA-GA**

**Scheme 1:**
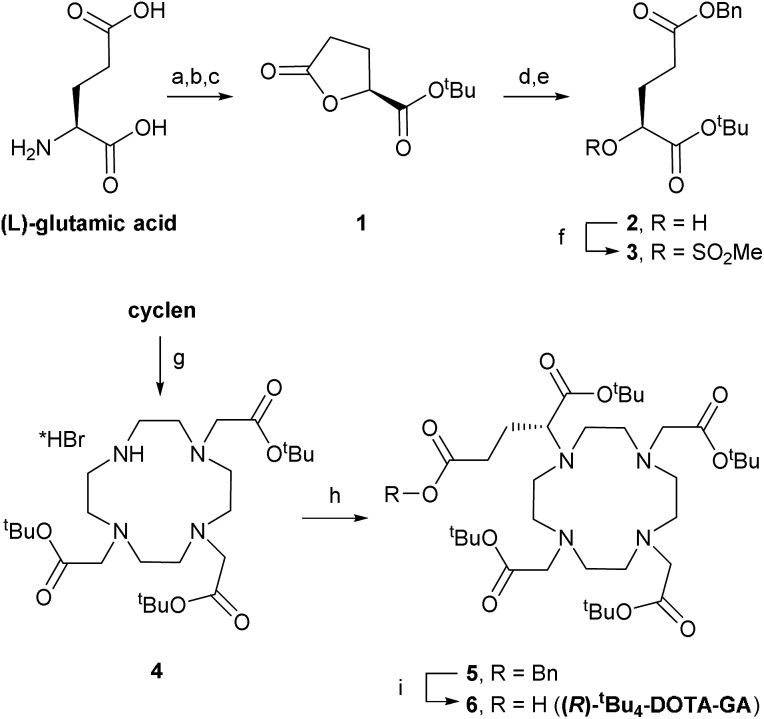
Synthesis of **(*R*)-*^t^*Bu_4_-DOTA-GA** (**6**). Reagents and conditions: (a) NaNO_2_, aq. HCl, dioxane, 0 °C → rt, 1.5 h; (b) (COCl)_2_, DMF, DCM, 0 °C → rt, 2 h; (c) ^*t*^BuOH, 2,6-lutidine, DCM, 0 °C → rt, 16 h, 38% over 3 steps; (d) 1 M KOH (1 equiv.), THF, rt, 2 h; (e) benzyl bromide, DMF, 8 h, 55% over 2 steps; (f) MsCl, DIPEA, DCM, 0 °C → rt, 30 min, 71%; (g) *tert*-butylbromoacetate, DMA, NaOAc, 0 °C → rt, 16 h, 70%; (h) **3**, K_2_CO_3_, MeCN, rt → 60 °C, 16 h, 70%; (i) H_2_, 5% Pd/C, MeOH, rt, 16 h, 80%.

DOTA-based building block **(*R*)-*^t^*Bu_4_-DOTA-GA** (**6**) was synthesised through adaptation of a literature procedure reported to give **6** in high purity and >97% enantiomeric excess (Scheme 1).^10^ l-Glutamic acid was converted to lactone **1** through diazotisation followed by *tert*-butyl ester formation, in moderate yield (over 3 steps, after crystallisation).^11^ Lactone ring opening with 1 equivalent of KOH, followed by benzylation of the intermediate potassium carboxylate, gave alcohol **2**, which was mesylated in good yield. Alcohol **2** was easy to store. Therefore, mesylate **3** was always prepared freshly before use.

Levy *et al.* synthesised benzyl-protected DOTA-GA **5** by mono-alkylation of cyclen with mesylate **3** followed by triple alkylation with *tert*-butylbromoacetate. Their procedure required 2 equivalents of cyclen (an expensive building) in the first step to avoid over-alkylation, and removal of excess cyclen after the reaction was essential to avoid problems in subsequent steps.^10^ In contrast, we isolated tri-functionalised cyclen **4** as its pure HBr salt by crystallisation from chloroform/diethyl ether.^12^ Alkylation of **4** with 1.2 equivalents of 3 under basic conditions afforded **5**.

Benzyl ester **5** was highly sensitive to trans-esterification under basic conditions. Even after filtration of the crude reaction mixture, sufficient potassium carbonate was present to give the corresponding methyl ester upon addition of methanol.^13^ Therefore, **5** was purified by automated reverse phase (RP; C18) chromatography before hydrogenolysis to afford **(*R*)-*^t^*Bu_4_-DOTA-GA** (**6**). We also noticed that amide bond formations with **6** are very sensitive to residual alcohol or water; rapid hydrolysis/methanolysis of activated esters of **6** is consistent with the sensitivity of **5** to trans-esterification. Therefore, **6** (1.35 g) was purified by automated RP (C18) chromatography and subsequent lyophilisation. The use of an acid-free eluent during this purification was crucial to avoid formation of salts, and for consistent results with subsequent amide bond formations. The ee of 6 was >97%, as determined by NMR analysis upon formation of amides with either enantiomer of α-methylbenzylamine, according to reported procedures.^10^

### Synthesis of trivalent Gd-DOTA reagents

**Scheme 2:**
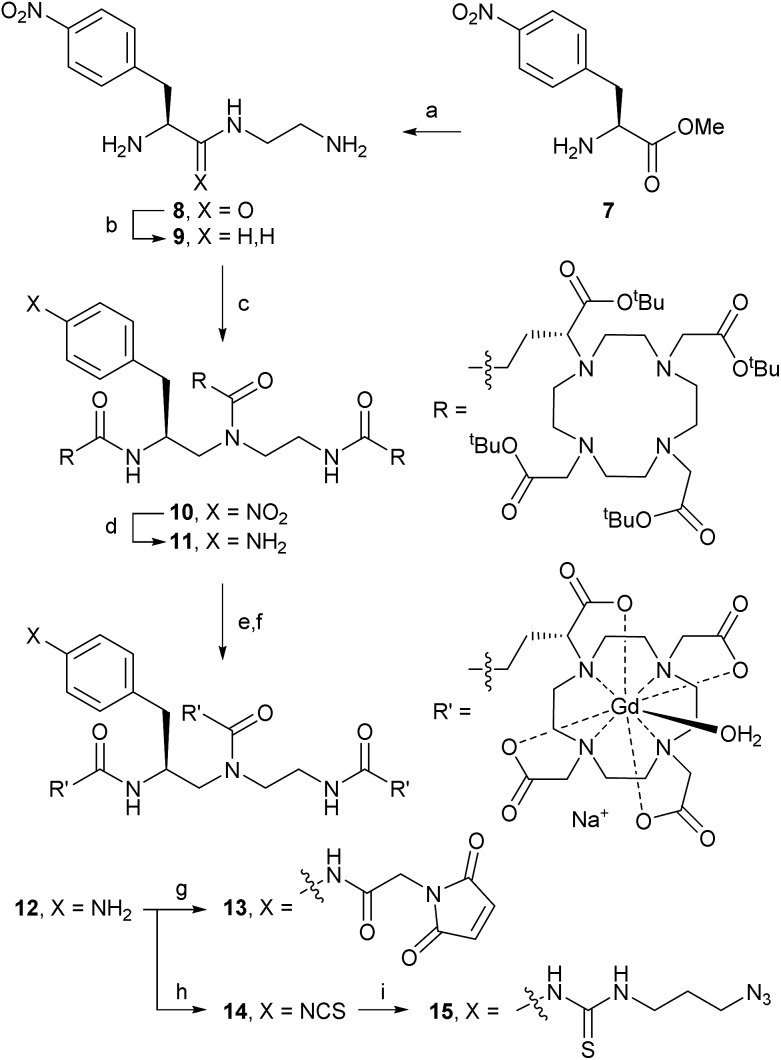
Synthesis of the trivalent Gd-DOTA reagents **[Gd-DOTA]_3_-maleimide** (**13**), **[Gd-DOTA]_3_-ITC** (**14**), and **[Gd-DOTA]_3_-N_3_** (**15**) *via* **[Gd-DOTA]_3_-NH_2_** (**12**). Reagents and conditions: (a) ethylene diamine, rt, 16 h, 97%; (b) BH_3_ in THF, 0 °C → reflux, 19 h, 43% (isolated as triple HCl salt); (c) **6**, HATU, DIPEA, DMF, 0 °C → rt, 16 h, 88%; (d) H_2_, 10% Pd/C, MeOH, 40 °C, 16 h, 93%; (e) TFA, TES, MsOH, 0 °C, 4.5 h; (f) GdCl_3_, aq. NaOH (pH 6.5), rt → 50 °C, 16 h, 75% over 2 steps; (g) 2-maleimidoacetic acid-OSu, HEPES buffer pH 7.3, DMSO, 6 h, 40%; (h) CSCl_2_, CHCl_3_ : H_2_O (1 : 1), rt, 16 h, 70%; (i) 3-azidopropylamine, carbonate buffer pH 9.4, DMSO, rt, 2 h, 76%.

Trivalent linker **9** was prepared in 2 steps from l-nitrophenylalanine methyl ester **7** according to a literature procedure (Scheme 2).^14^ Triamine **9** was isolated as its triple HCl salt, which is hygroscopic and needs to be stored under an inert atmosphere for consistent results in subsequent acylation with **(*R*)-*^t^*Bu_4_-DOTA-GA** (**6**). After extensive optimisation of reaction conditions, triple acylation of triamine **9** with **6** using HATU and DIPEA afforded **[(*R*)-*^t^*Bu_4_-DOTA]_3_-NO_2_** (**10**) in excellent yield. In contrast to reported procedures, only a small excess of **6** (1.1 equiv. per acylation) was required. The best conversion was seen with freshly freeze-dried **6** (purified as described above) and anhydrous DIPEA. Consistent with the patent literature, purification of **10** by flash chromatography was only partially successful, and gave low yield of pure material.^9^ However, straight-forward size exclusion chromatography (Sephadex LH-20), followed by lyophilisation, gave **10** in high yield.

Hydrogenation of the nitro group of **10** gave aniline **[(*R*)-*^t^*Bu_4_-DOTA]_3_-NH_2_** (**11**) as a pure compound, which was converted into **[Gd-DOTA]_3_-NH_2_** (**12**) in two steps. The use of methanesulfonic acid, in addition to TFA and the cation scavenger triethylsilane, was essential for the deprotection of all 12 *tert*-butyl esters of **11**.^15^ The reaction worked best when kept at or just above 0 °C; significant decomposition and reduced yields were observed if the reaction mixture was allowed to warm up. Therefore, the deprotected intermediate (as its methanesulfonate salt) was separated from the deprotection cocktail by precipitation upon addition of cold diethyl ether. The resulting trivalent DOTA ligand was then directly charged with Gd^3+^ to afford trivalent DOTA chelate **[Gd-DOTA]_3_-NH_2_** (**12**). The crude product **12** was purified by size exclusion chromatography to remove excess salts. Formation of the paramagnetic complex **12** was confirmed by high resolution ESI-MS.^15^ Purified aniline **12** was prepared in batches of *ca.* 350–400 mg, and could be stored at –20 °C for >1 year without signs of decomposition.

Next, aniline **12** was modified to install reactive handles for the labelling of targeting moieties, including peptides and biomolecules. Modifications of **12** were performed on small scales (6–50 mg) to produce material for bioconjugation studies. Acylation with the *N*-hydroxylsuccinimide ester of 2-maleimidoacetic acid (2-maleimidoacetic acid-OSu),^16^ afforded **[Gd-DOTA]_3_-maleimide** (**13**) suitable for selective modifications of thiols (*e.g.*, cysteines). Treatment of **12** with thiophosgene gave **[Gd-DOTA]_3_-ITC** (**14**) suitable for reactions with nucleophilic amines (*e.g.*, lysines). Isothiocyanate **14** could also be converted into the azide **[Gd-DOTA]_3_-N_3_** (**15**), suitable for, for example, copper-catalysed azide–alkyne cycloadditions (CuAAC) or strain-promoted azide–alkyne cycloadditions (SPAAC). 2-Maleimidoacetic acid-OSu and 3-azidopropylamine were separated from trivalent Gd-DOTA reagents **13** and **15**, respectively, using size exclusion chromatography before bioconjugation reactions; isothiocyanate **14** did not require purification.

### Bioconjugation studies with trivalent Gd-DOTA reagents

**Fig. 2:**
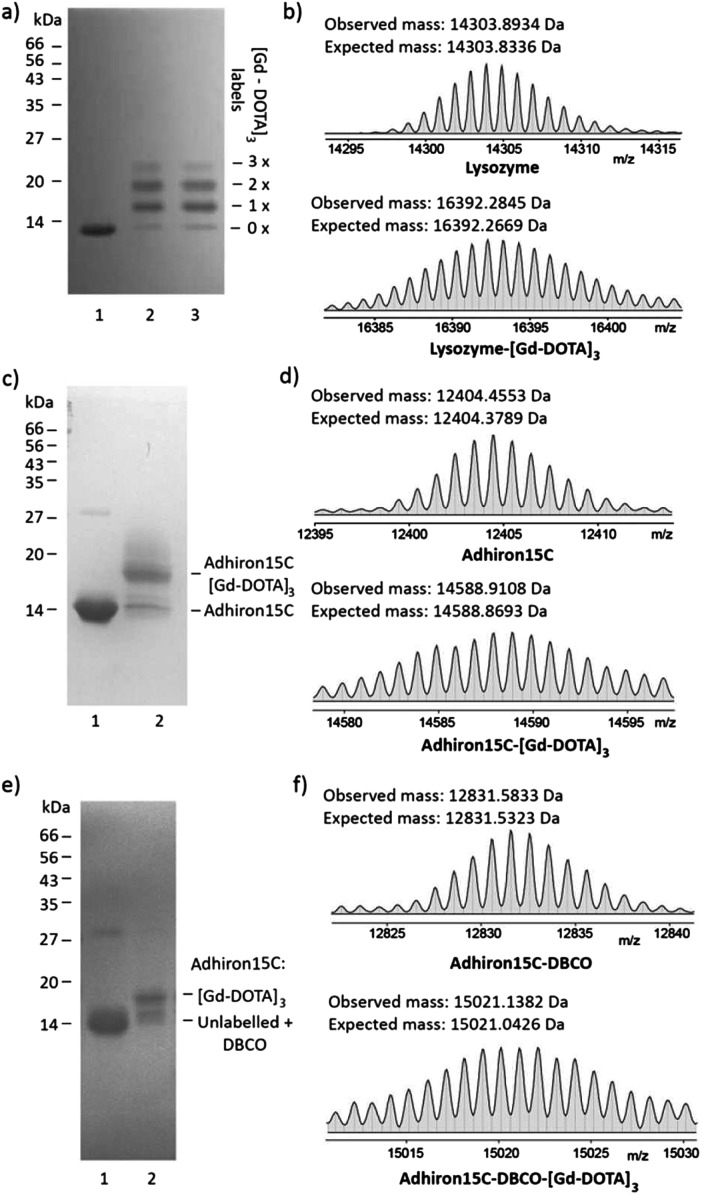
Modification of biomolecules with trivalent Gd-DOTA reagents. (a and b) Reaction of lysozyme (71 μM in 0.1 M Na_2_CO_3_) with ITC **14** (3 equiv.; 18 h) resulted in single, double and triple labelling, according to SDS-PAGE (a) and high resolution ESI-MS ((b); deconvoluted spectrum shown) analysis. In (a) lane 1: lysozyme; lane 2: lysozyme after reaction with ITC **14**; lane 3: ITC **14** pre-incubated with carbonate buffer for 45 min before reaction with lysozyme. (c and d) Reaction of **Adhiron15C** (84 μM in phosphate buffer pH 7.4) with maleimide **13** (40 equiv.; 6 h) resulted in single labelling only, according to SDS-PAGE (c) and high resolution ESI-MS ((d); deconvoluted spectrum shown) analysis. In (c) lane 1: **Adhiron15C** the minor band at higher mass represents a small amount of **Adhiron15C** dimer (disulfide); lane 2: **Adhiron15C** upon reaction with maleimide **13**. (e and f) Two-step labelling of **Adhiron15C** (50 μM in phosphate buffer pH 7.4) with azide **15** (2 equiv.; 6 h) resulted in rapid strain-promoted azide–alkyne cycloaddition according to SDS-PAGE (e) and high resolution ESI-MS ((f); deconvoluted spectrum shown) analysis. In (e) lane 1: **Adhiron15C** upon reaction with dibenzocyclo-octyne maleimide (**DBCO-Mal**; 20 equiv.; 6 h); lane 2: **Adhiron15C-DBCO** upon reaction with azide **15** (2 equiv.; 6 h).

Next, the suitability of trivalent Gd-DOTA reagents **13**, **14** and **15** for protein modification was investigated. Initially, bioconjugation experiments were performed with lysozyme. Incubation of lysozyme (71 μM in 0.1 M sodium carbonate) with three equivalents of **14** resulted in rapid bioconjugation (Fig. 2a and b). SDS-PAGE analysis showed mainly single and double labelling of lysozyme, and some triple labelling (Fig. 2a, lane 1). The identity of the singly- and doubly-labelled lysozyme was confirmed by ESI-MS (Fig. 2b and the ESI†).^17^ Preincubation of **14** in 0.1 M sodium carbonate for 45 minutes before addition of lysozyme resulted in identical labelling patterns (Fig. 2a, lane 2), which indicates that **14** is relatively stable in basic aqueous solution. In addition, **14** could be stored as a powder at –20 °C for >1 year without affecting its reactivity.

Our ongoing research focuses on the use of functionalised Adhirons for targeted imaging. Adhirons (also known as Affimers) are a novel class of small (*ca.* 12 kDa) antibody mimetics that can be selected rapidly *in vitro* against a range of targets using phage display protocols.^18^ Adhirons have excellent binding affinity and specificity, high thermal stability, low production costs and no disulfide bonds. Compared to antibodies, their much smaller size would facilitate more rapid tissue penetration and blood clearance, which is advantageous for *in vivo* imaging. In addition, the ease of engineering of site-specific modifications, including single or multiple cysteines, makes Adhirons well-suited for site-specific chemical labelling. To test the site-specific modification of Adhirons with trivalent Gd-DOTA reagents, bioconjugation reactions were performed with **Adhiron15C**, which contains a single cysteine on its C-terminus (pointing away from the binding loops) (Fig. 2c–f). **Adhiron15C** contains a C-terminal His tag and was purified by Ni nitrilotriacetic acid (NTA) affinity chromatography. Initially, labelling with maleimide **13** was performed directly in the elution buffer, at a protein concentration of 84 μM. SDS-PAGE analysis showed formation of a single labelling product even in the presence of 40 equiv. of **13** (Fig. 2c) and high resolution ESI-MS confirmed the identity of **Adhiron15C-[Gd-DOTA]_3_** (Fig. 2d).

Although the labelling of **Adhiron15C** with maleimide **13** was successful, a large excess of this precious reagent was needed. To check if inefficient labelling was caused by imidazole and/or tris(2-carboxyethyl)phosphine (TCEP) present in the Adhiron elution buffer, ligation reactions were also attempted after purification of **Adhiron15C** by dialysis into labelling buffer (PBS containing 10% glycerol and 0.05% Tween-20; pH 7.4) and/or gel filtration. However, this did not significantly improve the labelling reaction, and neither did the use of polymer-supported TCEP (instead of TCEP solution) to reduce disulfide bonds of Adhiron dimers before labelling. Therefore, a more efficient 2-step protocol for Adhiron labelling based on SPAAC^19^ was developed. Treatment of **Adhiron15C** (50 μM in labelling buffer) with the commercially available linker dibenzocyclo-octyne maleimide (**DBCO-Mal**; see the ESI† for structure; 20 equiv.) gave **Adhiron15C-DBCO** (Fig. 2f).

After buffer exchange (PD-10 column) and concentration, **Adhiron15C-DBCO** (89 μM in labelling buffer) was treated with azide **15** (2 equiv.), leading to rapid formation of **Adhiron15C-DBCO-[Gd-DOTA]_3_** by SPAAC (Fig. 2e and f). Although conversion was still not complete (most likely because of the sluggish maleimide ligation to form **Adhiron15C-DBCO**), this procedure gave similar conversion to direct ligation with maleimide **13**, but is significantly more efficient in terms of the required amount of trivalent Gd-DOTA reagent.

## Conclusions

Trivalent Gd-DOTA reagents have been developed for the site-specific functionalisation of (bio)molecules bearing amines, thiols, and/or (strained) alkynes. Detailed synthetic procedures have been reported, including recommendations regarding purification and storage to assure high-yielding reactions with sensitive intermediates. Procedures for the (site-specific) modification of different proteins were developed. Our reagents allow a range of chemoselective ligation reactions with targeting moieties, including biomolecules. Therefore, they may contribute to the development of novel MRI contrast agents targeted to proteins for which no suitable and/or easy-to-functionalise small molecule- or peptide-based binders are available, but for which phage display protocol can deliver antibody mimetics with high affinity and selectivity. In addition, our reagents could triple the gadolinium payload of previously described CTB-based neuronal tracers.^5^ We are currently using our reagents to optimise the gadolinium payload on Adhiron-based contrast agents, for example through multiple labelling or the inclusion of a polyvalent scaffold.^7^

## Experimental

### Synthetic procedures

The following compounds were prepared using literature methods and full reaction details can be found in the ESI:† **1**,^10^ **2**,^10^ **3**,^10^ **4**,^12^ **8**,^14^ **9**,^14^ 2-maleimidoacetic acid (**S1**)^16^ and 2-maleimidoacetic acid-OSu (**S2**).^16^

#### **(*R*)-*^t^*Bu_4_-DOTA-GA** benzyl ester **5**

A solution of benzyl 5-(*tert*-butoxy)-4-(methanesulfonyloxy)-5-oxopentanoate **3** (1.85 g, 4.9 mmol) in MeCN (20 mL) was added dropwise to a suspension of trialkylated cyclen **4** (2.4 g, 4.1 mmol) and potassium carbonate (1.7 g, 12.4 mmol) in MeCN (15 mL) at room temperature. The reaction mixture was stirred at 60 °C overnight. Once the reaction was complete according to LCMS, the reaction mixture was cooled, filtered over Celite, and concentrated *in vacuo*. The residue was taken up in DCM (20 mL), filtered over Celite, and concentrated *in vacuo*. The resulting orange oil was dissolved in MeCN : H_2_O (20 : 80) and purified by automated RP (C18) chromatography using gradient elution (MeCN–H_2_O–HCOOH, 15 : 90 : 0.1 to 85 : 10 : 0.1). The eluate was concentrated *in vacuo* to give an aqueous emulsion (6–10 mL) which was lyophilised to give the title compound (*as its double formic acid salt*) as a viscous amber oil (2.7 g, 70%). *ν*_max_ cm^–1^ 2979 and 1725; *δ*_H_ (500 MHz; CDCl_3_), 9.65–10.15 (br s, 2H), 8.48 (s, 2H), 7.30–7.38 (m, 5H), 5.11 (s, 2H), 3.71–3.79 (m, 4H), 3.44 (s, 2H), 3.34 (dd, *J* 4.2, 10.1, 1H), 3.12–3.22 (m, 8H), 2.94–3.00 (m, 4H), 2.85–2.94 (m, 4H), 2.38–2.52 (m, 2H), 2.03–2.11 (m, 1H), 1.86–1.94 (m, 1H), 1.44–1.47 (three s, 36H); *δ*_C_ (125 MHz; CDCl_3_), 172.5, 171.0, 169.4, 168.5, 166.0, 135.7, 128.6, 128.4, 128.3, 82.8, 82.4, 82.3, 66.4, 63.1, 56.4, 55.2, 52.9, 52.5, 49.9, 46.8, 30.9, 28.2, 28.1, 23.7; *m*/*z* HRMS (ESI) calcd for C_42_H_71_N_4_O_10_: 791.5165 [M + H]^+^, found 791.5174.

#### **(*R*)-*^t^*Bu_4_-DOTA-GA** **6**

5% Pd/C (250 mg) was added to a stirred solution of **5** (1.9 g, 2.4 mmol) in MeOH (40 mL) under an atmosphere of nitrogen. The reaction was then placed under an atmosphere of hydrogen (balloon; three cycles of evacuation (aspirator) and back-filling) and stirred overnight. Once the reaction was complete according to LCMS, the reaction mixture was filtered over Celite and the solvent removed *in vacuo*. The crude material was dissolved in MeCN : H_2_O (20 : 80) and purified by automated RP (C18) chromatography using gradient elution (MeCN–H_2_O, 10 : 90 to 90 : 10). The eluate was concentrated *in vacuo* to give an aqueous solution (5–10 mL) which was lyophilised to give the title compound as a colourless amorphous solid (1.35 g, 80%). *ν*_max_ cm^–1^ 3426, 3096, 2979, 1954 and 1720; *δ*_H_ (500 MHz; CDCl_3_) 6.62–6.97 (bs, 1H), 3.59–3.67 (m, 4H), 3.55 (t, *J* 6.9, 1H), 3.48 (s, 2H), 2.94–3.19 (m, 14H), 2.84–2.92 (m, 2H), 2.51–2.59 (m, 1H), 2.39–2.48 (m, 1H), 1.98–2.06 (m, 1H), 1.87–1.96 (m, 1H), 1.40–1.48 (three s, 36H); *δ*_C_ (125 MHz; CDCl_3_) 176.1, 171.4, 170.8, 170.3, 81.6, 81.3, 81.1, 63.6, 56.1, 55.7, 52.0, 51.7, 49.4, 33.1, 28.3, 28.2, 27.93, 27.87, 25.3; *m*/*z* HRMS (ESI) calcd for C_35_H_65_N_4_O_10_: 701.4695 [M + H]^+^, found 701.4702.

#### **[(*R*)-^t^Bu_4_-DOTA]_3_-NO_2_** **10**

DIPEA (526 μL, 3.20 mmol) was added to a cooled (0 °C), stirred solution of HATU (546 mg, 1.41 mmol), **6** (400 mg, 1.14 mmol), and 2-amino-3-(4′′-nitrophenyl) propyl (2′-aminoethyl)amine trihydrochloride **9** (55 mg, 0.32 mmol) in DMF (6 mL). The reaction mixture was stirred at 0 °C for 30 minutes, then allowed to warm to room temperature and stirred overnight. Once the reaction was complete according to LCMS, the reaction mixture was concentrated *in vacuo* and re-dissolved in MeOH, then purified by size exclusion chromatography (Sephadex LH-20; elution with MeOH). The purified product was concentrated *in vacuo*. The residue was taken up in H_2_O, then lyophilised to give the title compound as a colourless amorphous solid (650 mg, 88%). *δ*_H_ (500 MHz; CDCl_3_) 8.06–8.12 (m, 2H), 7.49–7.60 (m, 2H), 4.34–4.58 (m, 1H), 2.80–3.92 (m, 77H), 2.15–2.61 (m, 6H), 1.90–2.13 (m, 6H), 1.37–1.51 (m, 108H); *m*/*z* HRMS (ESI) calcd for C_116_H_207_N_16_O_29_: 763.1744 [M + 3H]^3+^, found 763.1739.

#### **[(*R*)-*^t^*Bu_4_-DOTA]_3_-NH_2_** **11**

10% Pd/C (180 mg) suspended in MeOH (1 mL) was added to a solution of **10** (410 mg, 0.18 mmol) in MeOH (5 mL) under an atmosphere of nitrogen. The reaction was then placed under an atmosphere of hydrogen (balloon; three cycles of evacuation (aspirator) and back-filling) and stirred at 40 °C until complete according to LCMS (16 h). The reaction mixture was cooled to room temperature, filtered over Celite, and concentrated *in vacuo* to give the title compound as a colourless amorphous solid (380 mg, 93%). *δ*_H_ (500 MHz; CDCl_3_) 6.91–7.02 (m, 2H), 6.53–6.62 (m, 2H), 4.31–4.21 (m, 1H), 1.63–3.92 (m, 89H), 1.39–1.50 (m, 108H); *m*/*z* (LC-ESI-MS) calcd for C_116_H_207_N_16_O_27_: 752.8 [M + 3H]^3+^, found 753.3 (pattern for sequential deprotection of ^*t*^Bu groups observed).

#### **[Gd-DOTA]_3_-NH_2_** **12** ^15^

Pre-cooled (0 °C) TFA (3.5 mL) and TES (1.2 mL, 30 mmol) were added to solid **11** (500 mg, 0.22 mmol), after which vigorous bubbling was observed. The reaction temperature was maintained at 0 °C for 20 minutes. Then, MsOH (227 μL) was added and the reaction mixture was stirred for 4 h at 0 °C. Once the reaction was complete according to LCMS, the reaction mixture was poured into cold ether (40 mL) in a falcon tube. The resulting precipitate was stored in the fridge for 1 hour then centrifuged (1800 × *g*, 5 min). The supernatant was decanted off and the residue dissolved in water, then lyophilised to give a colourless amorphous solid (510 mg). This solid was dissolved in H_2_O (4 mL) and the pH adjusted to 6.5 with 2 N NaOH. To this solution was added GdCl_3_·6H_2_O (3 × 90 mg, 0.73 mmol) successively in 3 portions; after each addition the pH was monitored until no further change was observed (*ca.* 20 min) and the pH readjusted to 6.5 with 2 N NaOH before the next addition. After the final addition, the pH was again adjusted to 6.5 and the cloudy pink solution was heated to 50 °C overnight. Once complete according to LCMS the mixture was filtered over Celite and lyophilised. The crude product was purified by size exclusion chromatography (Sephadex LH-20; elution with H_2_O). The purified product was lyophilised to give the title compound as a pale pink amorphous solid (360 mg, 75% over 2 steps). *m*/*z* HRMS (ESI, negative mode) calcd for C_68_H_98_Gd_3_N_16_O_27_: 681.1514 M^3–^, found 681.1494 (complex pattern due to Gd isotopes; the predicted and observed isotopic distributions were identical, see ESI†).

#### **[Gd-DOTA]_3_-maleimide** **13**

To a solution of **12** (50 mg, 0.024 mmol) in HEPES buffer (300 mM, pH 7.3, 300 μL) and DMSO (200 μL), a solution of 2-maleimidoacetic acid-OSu (18 mg, 0.072 mmol) in DMSO (100 μL) was added. Because a precipitate formed, the reaction mixture was centrifuged (17 000 × *g*, 1 min), and the supernatant was removed and retained before the pellet was redissolved in DMSO (100 μL) and added back to the supernatant. The reaction was spun (Stuart rotator) at room temperature for 4 h before a further aliquot of 2-maleimidoacetic acid-OSu (18 mg, 0.072 mmol) in DMSO (200 μL) was added. The reaction mixture was spun for a further 2 h, then loaded onto a size exclusion chromatography (LH_20_) column, and the product was eluted with H_2_O. The eluted product was lyophilised to give the title compound as an off-white solid (20 mg, 40%, some starting material **12** present according to LCMS), which was used immediately for bioconjugation reactions. *m*/*z* HRMS (ESI, negative mode) calcd for C_74_H_101_Gd_3_N_17_O_30_: 726.4889 M^3–^, found 726.4918 (complex pattern due to Gd isotopes; the predicted and observed isotopic distributions were identical, see ESI†).

#### **[Gd-DOTA]_3_-ITC** **14**

Thiophosgene (3.3 μL, 0.013 mmol) was added to a biphasic solution of **12** (50 mg, 0.024 mmol) in chloroform–H_2_O (50 : 50, 1 mL), and the reaction mixture was stirred vigorously overnight. Once the reaction was complete according to LCMS, the reaction mixture was diluted with water (3 mL) and the aqueous layer was extracted with chloroform (2 × 3 mL). The organic fractions were discarded and the remaining aqueous solution was lyophilised to give the title compound as a pale red amorphous solid (35 mg, 70%). *m*/*z* HRMS (ESI, negative mode) calcd for C_69_H_96_Gd_3_N_16_O_27_S: 695.1369 M^3–^, found 695.1346 (complex pattern due to Gd isotopes; the predicted and observed isotopic distributions were identical, see ESI†).

#### **[Gd-DOTA]_3_-N_3_** **15**

A solution of **14** (6.1 mg, 0.003 mmol) in H_2_O (169 μL) was dissolved in carbonate buffer (400 mM, pH 9.4, 134 μL) before a solution of 3-azidopropylamine (1.43 μL, 0.014 mmol) in DMSO (33 μL) was added. The reaction was spun (Stuart rotator) at room temperature for 2 h, then loaded onto a size exclusion chromatography (LH_20_) column, and the product was eluted with H_2_O. The purified product was lyophilised to give the title compound as an off-white solid (5 mg, 76%). *m*/*z* HRMS (ESI, negative mode) calcd for C_69_H_96_Gd_3_N_16_O_27_S: 728.4955 M^3–^, found 728.4938 (complex pattern due to Gd isotopes; the predicted and observed isotopic distributions were identical, see ESI†).

### Bioconjugation reactions

#### Labelling of lysozyme with **[Gd-DOTA]_3_-ITC** **14**

Hen egg white lysozyme at 71 μM in 0.1 M Na_2_CO_3_ (pH was not adjusted) was incubated at 25 °C with a 3-fold molar excess of **[Gd-DOTA]_3_-ITC** **14** for 18 h. For SDS-PAGE analysis, reactions were quenched by addition of one volume of 3× SDS-PAGE loading buffer (containing Tris buffer) and one volume of 150 mM DTT prior to heating to 100 °C for 5 minutes and analysis by SDS-PAGE and HRMS.

#### Site-specific labelling of **Adhiron15C** with **[Gd-DOTA]_3_-maleimide** **13**

The following stock solutions were prepared: **[Gd-DOTA]_3_-maleimide** **13** (10 mM in H_2_O); **Adhiron15C** (110 μM in elution buffer: 50 mM NaH_2_PO_4_, 500 mM NaCl, 300 mM imidazole, 10% glycerol, pH 7.4); tris(2-carboxyethyl)phosphine (TCEP; 50 mM in water). A solution of **Adhiron15C** (22.8 μL, 2.5 nmol), **13** (6 μL, 60 nmol) and TCEP (1.2 μL, 60 nmol) were mixed together and incubated at room temperature. After 3 h, a further aliquot of **13** solution (4 μL, 40 nmol) was added and the incubation continued for another 3 h. The reaction mixture was analysed by SDS-PAGE and HRMS.

#### Two-step site-specific labelling of **Adhiron15C** with **[Gd-DOTA]_3_-azide** **15**

A sample of **Adhiron15C** in elution buffer was dialysed (2× 1 : 1000) into labelling buffer (PBS containing 20% glycerol and 0.05% Tween-20; pH 7.4) to give a protein solution of 57 μM. 6.1 mL of this solution (0.35 μmol) was treated with TCEP in H_2_O (50 mM; 350 μL; 17.5 μmol), labelling buffer (185 μL) and **DBCO-Mal** in DMSO (20 mM; 350 μL, 7 μmol) to give a final protein concentration of 50 μM. The reaction was rocked for 6 hours and monitored by mass spectrometry. Upon completion, the material was passed through a buffer exchange column (PD-10, GE Healthcare) according to manufacturer's instructions, eluting 0.5 mL fractions with labelling buffer. Fractions containing protein were identified by BioRad colourimetric assay and pooled. The protein was then concentrated to 89 μM by spin concentrator (3 kDa cut-off), analysed by HRMS, and used immediately in the next step (or flash frozen and stored at –80 °C if required).

2 mL of this **Adhiron15C-DBCO** solution (0.17 μmol) was treated with **[Gd-DOTA]_3_-azide** **15** (2 mM in H_2_O; 175 μL; 0.35 μmol) and the solution was rocked for 6 hours. Upon completion, the material was passed through a buffer exchange column (PD-10, GE Healthcare) according to manufacturer's instructions, eluting 0.5 mL fractions with labelling buffer. Fractions containing protein were identified by BioRad colourimetric assay and pooled. **Adhiron15C-DBCO-[Gd-DOTA]_3_** was concentrated to 323 μM by spin concentrator (3 kDa cut-off), analysed by SDS-PAGE and HRMS, flash frozen and stored at –80 °C.
