# Supplementary material for: Trivalent Gd-DOTA reagents for modification of proteins
Source: RSC Adv. 2015 Nov 11;5(116):96194–200. doi: 10.1039/c5ra20359g (PMC4786947; doi:10.1039/c5ra20359g)
Supplement: Supplementary file 1 [file RA-005-C5RA20359G-s001.pdf]

Electronic Supplementary Material (ESI) for RSC Advances.
This journal is © The Royal Society of Chemistry 2015

Supporting Information

Trivalent Gd-DOTA reagents for modification of proteins

Martin J. Fisher,[ab] Daniel J. Williamson,[ab] George M. Burslem,[ab] Jeffrey P. Plante,[ab] Iain W. Manfield,[bc] Christian Tiede,[bc] James R. Ault,[bc] Peter G. Stockley,[bc] Sven Plein,[de] Azhar Maqbool,[de] Darren C. Tomlinson,[bc] Richard Foster,[abc] Stuart L. Warriner,[ab] and Robin S. Bon*[abc]

a) *School of Chemistry, University of Leeds, LS2 9JT, UK; r.bon@leeds.ac.uk.*
b) *Astbury Centre for Structural Molecular Biology, University of Leeds.*
c) *School of Molecular and Cellular Biology, University of Leeds.*
d) *Leeds Institute of Cardiovascular and Metabolic Medicine, University of Leeds*
e) *Multidisciplinary Cardiovascular Research Centre, University of Leeds*

Contents

1. Synthesis and characterisation of compounds 2
2. Materials and methods for bioconjugations 6
3. Copies of spectra of new compounds/compounds made by new procedures 8
4. Additional mass spectrometry data for labelled lysozyme 14
5. References 18

1. Synthesis and characterisation of compounds

General Remarks

Air- and/or moisture-sensitive reagents were handled under an atmosphere of dry nitrogen, using solvents from a solvent purification system (Innovative Technology Inc. PureSolv®). Anhydrous DMF was purchased from Sigma-Aldrich and used as provided.

Flash column chromatography was carried out using silica (35–70 μm particles) according to the method described by Still, Kahn and Mitra.[1] Thin layer chromatography was carried out on commercially available pre-coated aluminium plates (Merck silica 2 8 8 0 Kieselgel 60F254). Lyophilisation of compounds was performed using a Virtis Benchtop K freeze dryer. Size exclusion chromatography (SEC) was carried out using columns packed with Sephadex® LH-20 resin (Sigma), with the following column dimensions: height 55 cm, diameter 1.6 cm, packed with 76 mL of LH-20 resin for sample sizes below 50 mg; height 100 cm, diameter 2.8 cm, packed with 492 mL of LH-20 resin for sample sizes above 50 mg. An autosampler (LKB BROMMA, 2211 SUPERRAC) combined with a peristaltic pump (WATSON-MARLOW, 101U) was used to collect timed fractions at either 2 min (small SEC column) or 12 min (large SEC column) intervals. Elution rate was between 0.3 mL/min and 1 mL/min, depending on column size, sample size and solvent (MeOH or H_2O). Automated RP (C18) chromatography was performed using a Biotage Isolera™ Prime Advanced Flash Purification system with RediSep Rf C18 columns (26 g or 130 g; Teledyne Isco). Compounds were eluted with the indicated solvent mixtures/gradients and elution was monitored by UV detection, and fractions were analysed by LC-ESI-MS before combination and concentration of fractions containing pure product.

Proton and carbon NMR spectra were recorded on a Bruker Advance DPX 300, Advance 500 or DRX500 spectrophotometer using an internal deuterium lock. Carbon NMR spectra were recorded with composite pulse decoupling using the waltz 16 pulse sequence. Chemical shifts are quoted in parts per million downfield of tetramethylsilane and referenced to residual solvent peaks ($CDCl_3$: ^1H = 7.26 ppm, ^{13}C = 77.16 ppm, CD_3OD: ^1H = 3.31 ppm, ^{13}C = 49.00 ppm) and coupling constants (*J*) are given in Hz. NMR spectra were recorded at 500 K unless otherwise stated. ^1H NMR spectra have been provided for all novel compounds and compounds made by procedures for which no NMR has previously been published; for all other compounds a reference has been provided. For compounds containing gadolinium mass spectra have been provided. Mass spectrometry was performed on a Waters Synapt HDMS quadrupole-IMS-time-of-flight instrument using Z-spray nanoelectrospray ionisation; high resolution electrospray MS (ESI^+ or ESI^-) was performed on a Bruker Daltonics MicroTOF mass spectrometer or on a Bruker MaXis Impact QqTOF mass spectrometer. Infrared spectra were recorded on a Perkin Elmer Fourier-Transfer spectrometer.

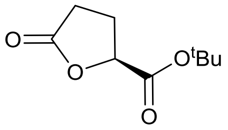

Tert-butyl 5-oxooxolane-2-carboxylate 1[2] A solution of $NaNO_2$ (8.4 g, 122 mmol) in water (20 mL) was added dropwise over 4 hours, using a syringe pump, to a stirred solution of L-glutamic acid (12 g, 81 mmol) in dioxane (9 mL) and 4.8M HCl (49 mL) at 0 ˚C. Upon completion of the addition, the reaction mixture was allowed to warm to room temperature and stirred for a further 1.5 h. Once complete, the solvents were removed in vacuo and the residue lyophilised to remove the last traces of water. The dried solids were taken up in EtOAc (100 mL), dried

($MgSO_4$) and filtered, and the filter cake was washed with EtOAc (50 mL). The solvents were removed in vacuo to give the title compound as a viscous, heterogeneous oil (12.3 g, contaminated with EtOAc according to ^{1}H NMR). This material was dissolved in DCM (30 mL) containing DMF (200 µM) and the solution was cooled to 0˚C. Then, oxalyl chloride (9.6 mL, 115 mmol) was added dropwise at 0 ˚C. Once addition was complete, the reaction was allowed to warm to room temperature and stirred for 2 h. The solvents were removed in vacuo to give the intermediate acid chloride as a crude brown oil, which was added dropwise to a cooled solution (0 ˚C) of tert-butanol (8 mL, 75 mmol) and 2,6 lutidine (5.3 mL, 46 mmol) in DCM (34 mL). After addition was complete, the reaction was allowed to warmed to room temperature and stirred overnight. The reaction mixture was then diluted with DCM (100 mL) and washed with 10% citric acid (100 mL), water (100 mL), saturated sodium bicarbonate (100 mL) and brine (50 mL). The organic layer was concentrated in vacuo. The residue was taken up in EtOAc (50 mL), loaded onto a plug consisting of celite (bottom), charcoal (middle) and silica (top), and eluted with EtOAc. Fractions containing the acid were concentrated in vacuo to give a light brown oil which was crystalised from EtOAc—Hexane (5:95, 10 mL) in the fridge overnight to give the title compound (2.8 g, 38%). δ_H (500 MHz; $CDCl_3$) 4.83 (dd, *J* 8.0, 4.1, 1H), 2.48–2.68 (m, 3H), 2.26–2.32 (m, 1H), 1.52 (s, 9H); δ_C (75 MHz; $CDCl_3$) 176.2, 169.0, 83.1, 76.3, 27.9, 26.8, 25.9; *m/z* (LC-ESI-MS) 209.1 $[M + Na]^+$ 100%; HRMS (ESI) calcd. for $C_9H_{14}NaO_4$: 209.0784 $[M + Na]^+$, found 209.0785.

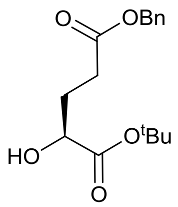

Benzyl 5-(tert-butoxy)-4-hydroxy-5-oxopentanoate 2[2] A 1M KOH solution (11.8 mL) was added in one portion to a cooled solution (0 ˚C) of **1** (2.2 g, 11.8 mmol) in THF (8.5 mL). The reaction mixture was stirred for 10 min, then allowed to warm to room temperature for 2 h, when the reaction was complete according to LCMS. Then, the reaction mixture was concentrated in vacuo to give a crude salt which was dried under high vacuum overnight. The resulting residue (2.2 g) was suspended in DMF and benzyl bromide (1.4 mL, 11.8 mmol) was added dropwise. The reaction mixture was stirred for 8 h at room, when the reaction was complete according to LCMS. Then, the reaction mixture was poured into water (50 mL), extracted with EtOAc (3 × 50 mL), and the combined organic layers were washed with brine (50 mL), dried ($MgSO_4$) and concentrated in vacuo. The crude material was purified by column chromatography eluting with EtOAc—hexane (10:90) to give the title compound as a colourless oil (1.9 g, 55%). δ_H (500 MHz; $CDCl_3$) 7.33–7.44 (m, 5H), 5.16 (s, 2H), 4.10–4.14 (m, 1H), 2.91 (d, *J* 5.4, 1H), 2.47–2.62 (m, 2H), 2.15–2.23 (m, 1H), 1.89–1.98 (m, 1H), 1.52 (s, 9H); δ_C (75 MHz; $CDCl_3$) 173.9, 173.1, 135.9, 128.6, 128.3, 128.2, 82.9, 69.7, 66.4, 29.8, 29.6, 28.0; *m/z* (LC-ESI-MS) 317.1 $[M + Na]^+$ 100%; HRMS (ESI) calcd. for $C_{16}H_{22}NaO_5$: 317.1357 $[M + Na]^+$, found 317.1359.

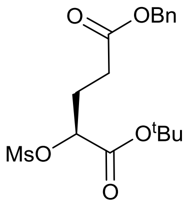

Benzyl 5-(tert-butoxy)-4-(methanesulfonyloxy)-5-oxopentanoate 3[2] Methanesulfonyl chloride (550 µL, 7.1 mmol) was added dropwise to a cooled solution (0 ˚C) of **2** (1.9 g, 6.5 mmol) and DIPEA (1.38 mL, 8.4 mmol) in DCM (70 mL). Once addition was complete, the reaction mixture was allowed to warm to room temperature and stirred for 30 min. Once the reaction was complete according to LCMS, the reaction mixture was poured into water (25 mL). The organic layer was separated, washed with brine, dried ($MgSO_4$) and concentrated in vacuo. The last traces of MsCl were removed under high vacuum on a rotary evaporator at 50 ˚C to give the title compound as a colourless oil (1.9 g, 71%). The

compound was used immediately in the synthesis of (*R*)-^{t}Bu$_4$-DOTA-GA benzyl ester **5**. δ_H (500 MHz; $CDCl_3$) 7.35–7.41 (m, 5H), 5.17 (s, 2H), 5.00 (dd, *J* 8.6, 4.1, 1H), 3.14 (s, 3H), 2.54–2.61 (m, 2H), 2.31–2.38 (m, 1H), 2.15–2.23 (m, 1H), 1.52 (s, 9H); *m/z* (LC-ESI-MS) 395.1 $[M + Na]^+$ 100%.

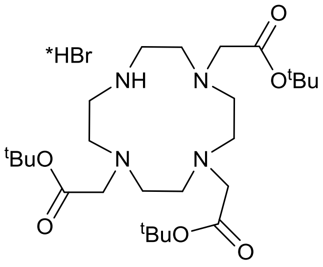

***tert*-Butyl 2′-(4,7-bis(2′-(tert-butoxy)-2′-oxoethyl)-1,4,7,10-tetraazacyclododecan-1-yl)acetate hydrobromide 4**[3] A solution of tert-butyl bromoacetate (18.7 g, 96 mmol) in DMA (20 mL) was added dropwise over 30 minutes to a cooled suspension (0 ˚C) of cyclen (5.0 g, 29 mmol) and NaOAc (7.9 g, 96 mmol) in DMA (60 mL). Then, the reaction mixture was allowed to warm to room temperature and stirred overnight. Once the reaction was complete according to LCMS, the reaction mixture was poured into water (300 mL) to give a clear solution. Then $KHCO_3$ (15 g, 180 mmol) was added portion-wise and the resulting suspension was filtered. The filter cake was dissolved in chloroform (250 mL), washed with water (100 mL) and dried ($MgSO_4$). The chloroform solution was reduced in volume to approximately 20 mL under reduced pressure. Then, ether (250 mL) was added to the remaining solution and the mixture allowed to stand for 30 minutes. The resulting crystals were collected by filtration and dried in vacuo to give the title compound as colourless plates (12.1 g, 70%). ν_{max}/cm^{-1} 2950, 2735, 1719 and 1577; δ_H (500 MHz; $CDCl_3$) 9.90–10.19 (bs, 1H), 3.38 (s, 4H), 3.29 (s, 2H), 3.06–3.14 (m, 4H), 2.84–2.99 (m, 12H), 1.43–1.49 (m, 27H); δ_C (125 MHz; $CDCl_3$) 170.5, 169.6, 81.9, 81.7, 58.2, 51.3, 51.2, 49.2, 48.7, 47.6, 28.23, 28.20; *m/z* (LC-ESI-MS) 537.4 $[M + Na]^+$ 100%; HRMS (ESI) calcd. for $C_{26}H_{50}N_4NaO_6$: 537.3622 $[M + Na]^+$, found 537.3627.

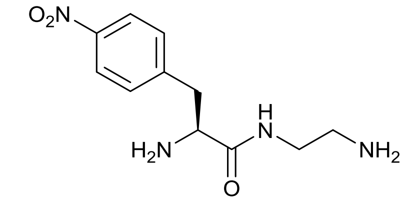

2-Amino-*N*-(2′-aminoethyl)-3-(4″-nitrophenyl)propanamide 8[4] (*S*)-(+)-4-nitrophenylalanine methyl ester (3.0 g, 13.4 mmol) was added to a solution of neat ethylene diamine (18 mL, 270 mmol) in 250 mg portions every 10 minutes. After addition was complete, the mixture was stirred at room temperature overnight. Once the reaction was complete according to LCMS, the excess ethylene diamine was removed in vacuo; the the last traces were removed by repeated azeotropic distillation with toluene. The residue was diluted with 7 M ammonium hydroxide solution (30 mL) and the pH was adjusted to 14 using 2N NaOH. The aqueous phase was extracted with DCM (8 × 30 mL), the combined organic extracts dried ($MgSO_4$), and the solvent removed in vacuo to give the title compound as an orange-yellow oil (3.3 g, 97%). ν_{max}/cm^{-1} 3400 (broad), 2934, 1652 and 1515; δ_H (300 MHz; $CDCl_3$) 8.17 (d, *J* 8.7, 2H), 7.50–7.90 (bs, 1H), 7.40 (d, *J* 8.7, 2H), 3.67 (dd, *J* 4.4, 8.7, 1H), 3.26–3.38 (m, 3H), 2.93 (dd, *J* 8.6, 13.6, 1H), 2.81 (t, *J* 6.0, 2H), 1.35 (s, 4H); δ_C (75 MHz; $CDCl_3$) 173.6, 150.0, 145.9, 130.2, 123.8, 56.2, 41.8, 41.4, 41.0; *m/z* (LC-ESI-MS) 253.1 $[M + H]^+$ 100%; HRMS (ESI) calcd. for $C_{11}H_{17}N_4O_3$: 253.1295 $[M + H]^+$, found 253.1304.

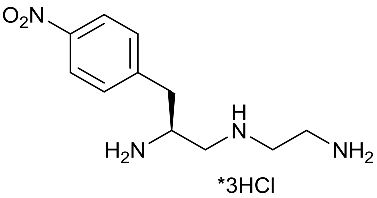

2-Amino-3-(4″-nitrophenyl) propyl (2′-aminoethyl)amine trihydrochloride 9[4] 1M BH_3 THF (45 mL) was added dropwise to a cooled solution (0 ˚C) of **8** (1.15 g, 4.6 mmol) in anhydrous THF (46 mL). Then, the reaction mixture was allowed to warm to room temperature, stirred for 3 hours, and

then heated to reflux overnight. Once the reaction was complete according to LCMS, the reaction mixture was cooled to 0 °C, quenched with water and concentrated to dryness in vacuo. The crude solid was taken up in 5N HCl (60 mL) and the mixture was heated to reflux for 2.5 h, then concentrated to dryness in vacuo. The residue was dissolved in concentrated aqueous ammonia (50 mL) and water (50 mL) and adjusted to pH 14 with 2N NaOH solution. The aqueous layer was extracted with DCM (150 mL) using continuous liquid-liquid extraction for 5 h. The organic layer was dried ($MgSO_4$) and concentrated in vacuo to give a dark orange oil. This oil was dissolved in ethanol (4 mL), and precipitation was induced by dropwise addition of 2N HCl in ether (1 mL). The resulting suspension was stirred at 50 °C for 5 h. The precipitate was collected by filtration and washed with ether to give the title compound as an orange-yellow amorphous solid (470 mg, 43%). ν_{max}/cm^{-1} 2979, 1728 and 1519; δ_H (500 MHz; CD_3OD) 8.32 (d, *J* 8.8, 2H), 7.73 (d, *J* 8.8, 2H), 4.17–4.24 (m, 1H), 3.60 (dd, *J* 13.7, 7.5, 1H), 3.48–3.56 (m, 5H), 3.45 (dd, *J* 14.6, 6.6, 1H), 3.26 (dd, *J* 14.6, 8.4, 1H); δ_C (125 MHz; MeOD) 149.2, 143.3, 132.0, 125.3, 51.2, 50.4; 46.7, 37.7, 37.1; *m/z* (LC-ESI-MS) 239.1 $[M + H]^+$ 100%; HRMS (ESI) calcd. for $C_{11}H_{18}N_4O_2$: 239.1503 $[M + H]^+$, found 239.1507.

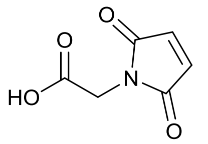

2-maleimidoacetic acid (S1)[5] Maleic anhydride (6.6 g, 66 mmol) and glycine (5 g, 66 mmol) were stirred in acetic acid (80 mL) at room temperature for 3 h. The resulting suspension was filtered under reduced pressure to obtain a solid that was washed with ice water (10 ml) before being dried in vacuo overnight to give maleic carboxymethylamide as a white solid (10 g, 87%) . Without further purification, the solid (5.1 g) was added to a stirred solution of Et_3N (9.2 mL) in toluene (500 mL) before the reaction mixture was heated to reflux for 3 h using a Dean-Stark apparatus. The reaction mixture was left to cool to room temperature, and the solution decanted and concentrated. The resulting solid was dissolved in water (100 mL) and the pH of the solution adjusted to pH 3 using 1N HCl. The product was extracted with EtOAc (3 × 300 mL), dried ($MgSO_4$) and concentrated in vacuo to give the title compound as an amorphous solid (1 g, 23%). δ_H (500 MHz,CD_3OD) 7.90 (s, 2H), 4.22 (s, 2H); δ_C (125 MHz, CD_3OD) 171.7, 171.0, 135.7, 39.3; HRMS (ESI) calcd. for $C_6H_4NO_4$: 154.0146 $[M - H]^-$, found 154.0138.

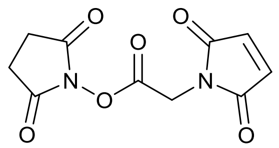

2-maleimidoacetic acid-OSu (S2)[5] Compound **S1** (300 mg, 1.9 mmol) and N-hydroxysuccinimide (452 mg, 3.9 mmol) were dissolved in anhydrous THF (30 mL). A solution of DCC (803 mg, 3.9 mmol) in anhydrous THF (30 mL) was added dropwise. The resulting suspension was stirred overnight at room temperature. Then, 4 drops of acetic acid were added and the mixture was stirred for a further 1 h. The reaction mixture was filtered, the filtrate concentrated and re-suspended in 2-propanol (100 mL) for 1h with stirring. The suspension was filtered, and the resulting solid was washed with 2-propanol (30 mL) and dried in vacuo to leave the title compound as an off-white amorphous solid (0.464 g, 95%). δ_H (500 MHz,CD_3OD) 7.20 (s, 2H), 4.72 (s, 2H), 2.81 (s, 4H, s); δ_C (125 MHz, CD_3OD) 169.7, 169.7, 164.4, 135.2, 36.3, 25.3; HRMS (ESI) calcd. for $C_{10}H_8N_2O_6Na$: 275.0274 $[M + H]^+$, found 275.0273.

2. Materials and methods for bioconjugations

Structure of DBCO maleimide

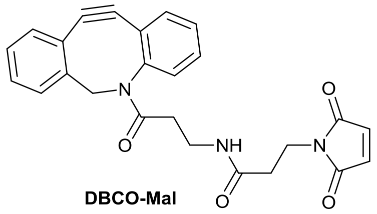

Buffers

All solutions were made up with 18.2 MΩ purified water and the pH of the solutions was adjusted using 1M NaOH and/or 4M HCl.

Phosphate-buffered saline (PBS, 1 L, pH 7.4): 137 mM NaCl (8 g), 2.7 mM KCl (0.2 g), 10 mM Na_2HPO_4 (1.44 g), 1 mM $CaCl_2 \cdot 2H_2O$ (0.133 g) and 0.5 mM $MgCl_2 \cdot 6H_2O$ (0.10 g)

Tris buffer (200 mM, 10 mL, pH 7.5): 200 mM Tris·HCl (0.314 g)

5× SDS-PAGE running buffer (10 mL): 125 mM tris base (0.156 g), 960 mM glycine (0.72 g) and 0.5% (*w/v*) SDS

Sodium dodecyl sulfate-polyacrylamide gel electrophoresis (SDS-PAGE)

A BioRad tetragel apparatus was used to determine the purity and size of proteins. A resolving gel of appropriate percentage was prepared using the materials listed in Table 1 – 14% for the gel in Figure 1a; 15% for the gel in Figure 1d). After the tetramethylethylenediamine (TEMED) was added the solution was thoroughly mixed and immediately added to the apparatus before 1-butanol (0.5 mL) was loaded onto the top of the gel. Once the resolving gel had set the 1-butanol was carefully removed and a stacking gel (5%) was prepared using the materials listed in **Table S1**. After the TEMED was added the solution was thoroughly mixed and immediately applied to the top of the resolving gel. A comb with a suitable number of lanes was inserted into the stacking gel layer before it set.

	**Resolving gel Volume**			**Stacking gel Volume**
**Components**	10%	12%	15%	5%
H_2O	4 mL	3.2 mL	2.3 mL	2.45 mL
30 % acrylamide/bisacrylamide mix (37:5:1)	3.3 mL	4 mL	5 mL	0.67 mL
1.5 M Tris (pH 8.8)	2.5 mL	2.5 mL	2.5 mL	N/A
1 M Tris (pH 6.8)	N/A	N/A	N/A	0.75 mL
10% sodium dodecyl sulfate (SDS)	0.1 mL	0.1 mL	0.1 mL	0.04 mL
20% or 10% ammonium persulfate (APS)	0.1 mL	0.1 mL	0.1 mL	0.04 mL
tetramethylethylenediamine (TEMED)	5 µL	5 µL	5 µL	5 µL

Table S1. Polyacrylamide gel components for SDS-PAGE analysis

Once the gel had set it was sequestered inside an electrophoresis tank, submerged in an optimum volume of SDS running buffer and the comb was removed. Protein samples (10 µL) were mixed with an equal volume of loading buffer, heated to 95 °C for 10 minutes and

applied to the lanes on the gel. Electrophoresis was performed at 180 V until the blue loading buffer could be seen to reach the bottom of the gel. The gel was submerged in Instant Blue Stain (TripleRed) for 30 minutes to visualise the protein bands.

Adhiron15C

Adhiron15C was based on a sequence selected by phage display, using an inflammatory marker as target, according to procedures reported by Tiede et al.[6] **Adhiron15C** , which contains a single cysteine in the C-terminus (...A**C**AAAHHHHHHHH), was purified using Ni nitrilotriacetic acid (NTA) affinity chromatography, eluting with elution buffer: 50 mM NaH_2PO_4, 500 mM NaCl, 300 mM imidazole, 10% glycerol, pH 7.4. Labelling was performed in this elution buffer.

3. Copies of spectra of new compounds/compounds made by new procedures

(*R*)-^{t}Bu$_4$-DOTA-GA benzyl ester 5.

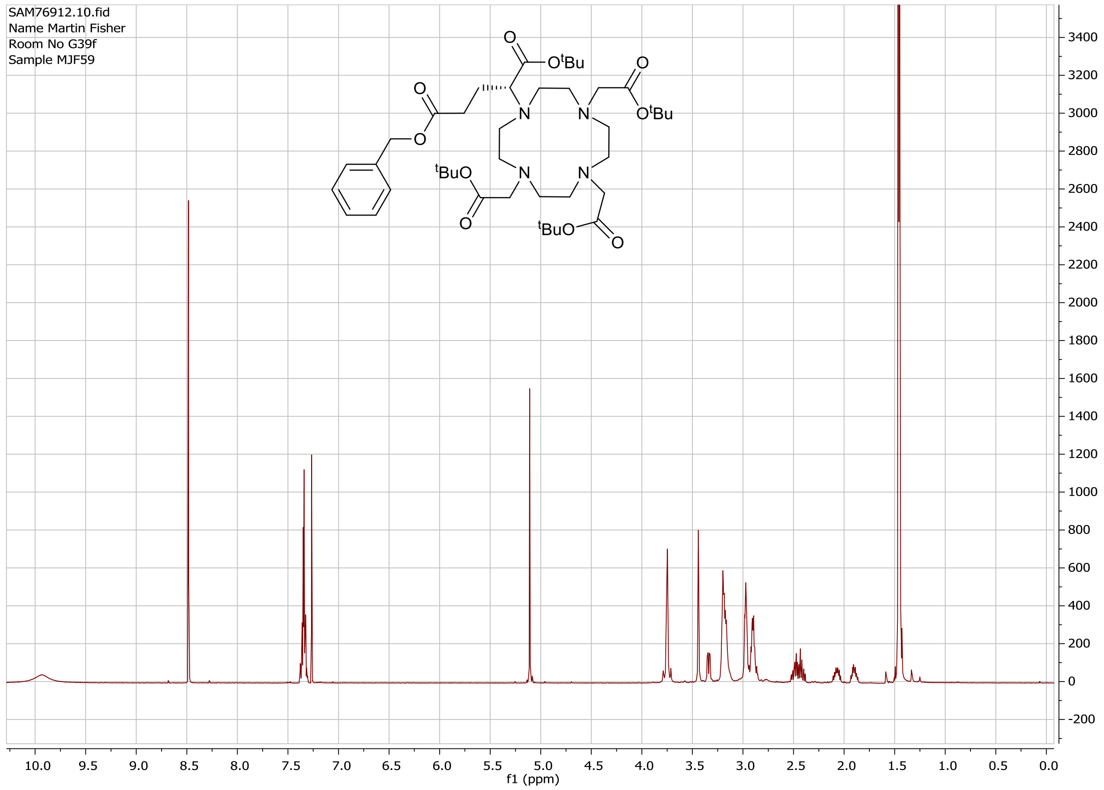

(*R*)-^{t}Bu$_4$-DOTA-GA 6.

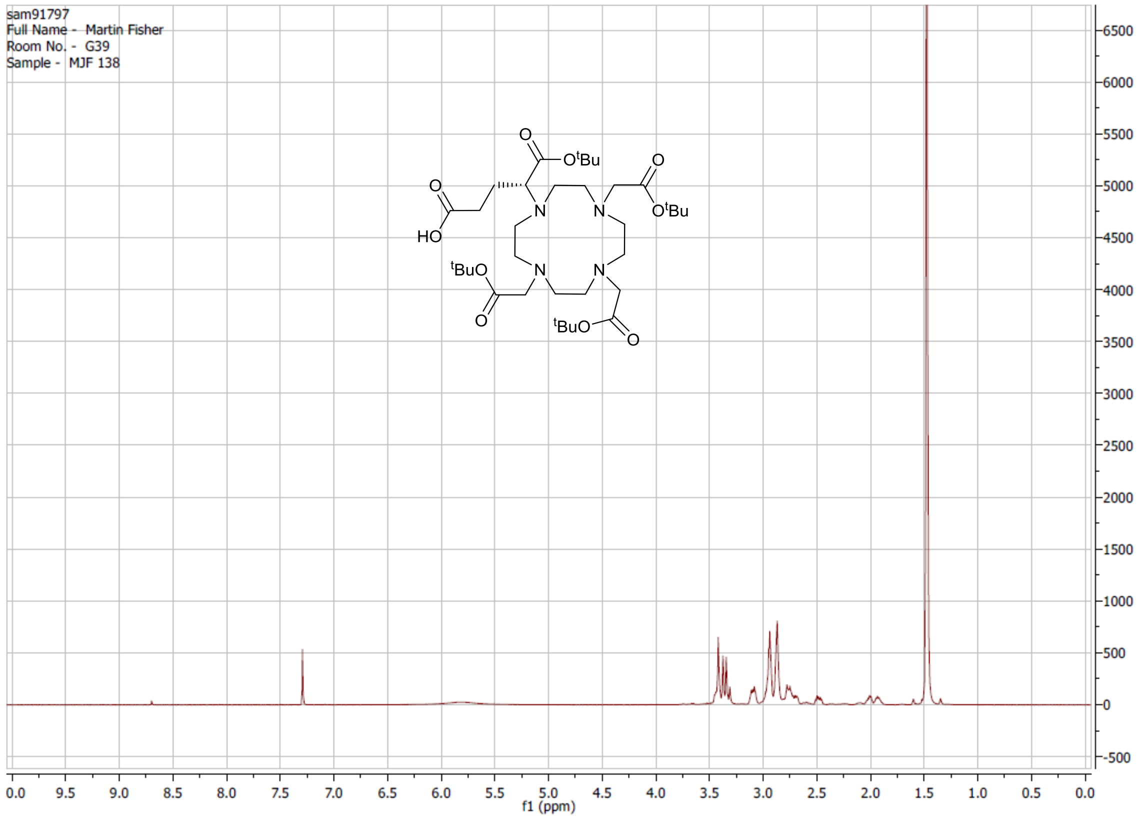

[(*R*)-^{t}Bu$_4$-DOTA]$_3$-NO$_2$ 10.

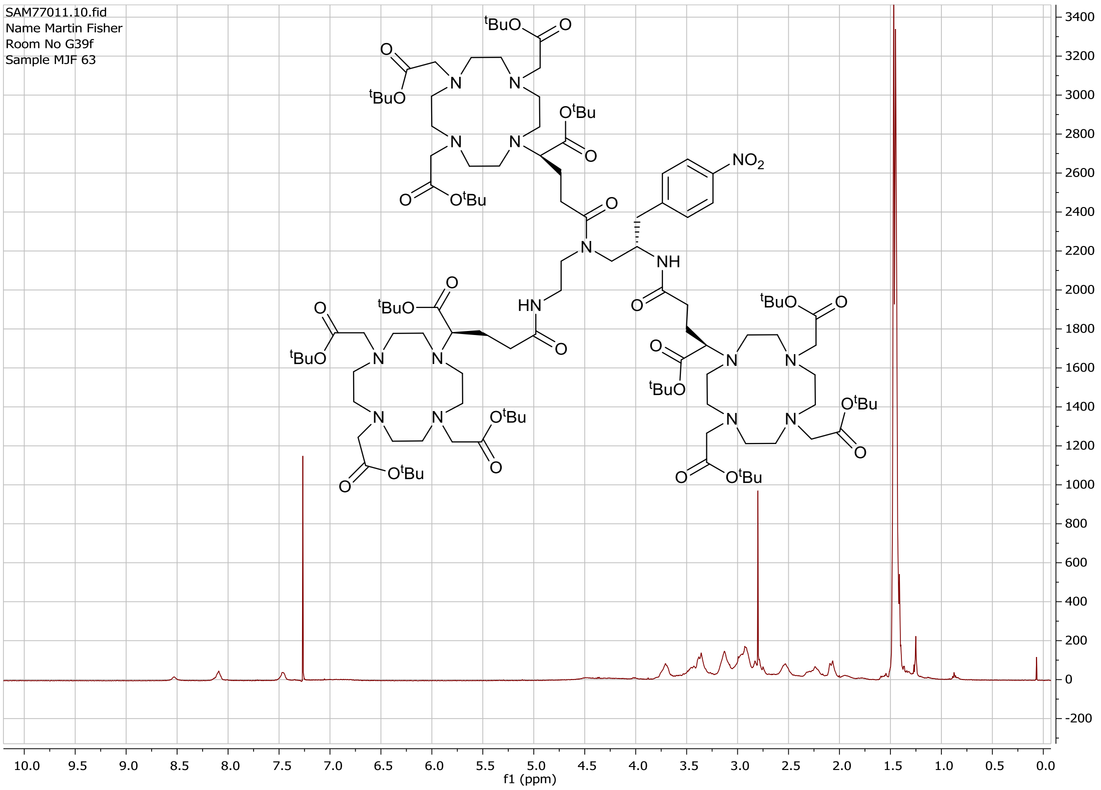

[(*R*)-^{t}Bu$_4$-DOTA]$_3$-NH$_2$ 11.

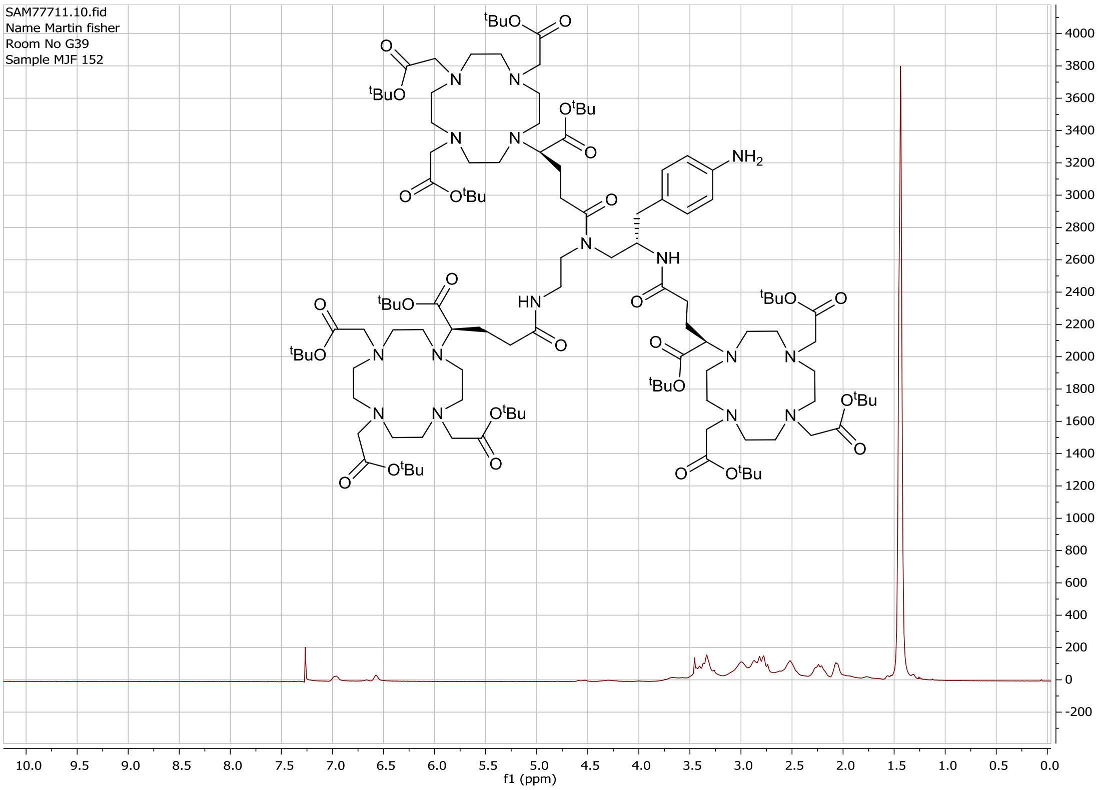

[Gd-DOTA]$_3$-NH$_2$ 12.

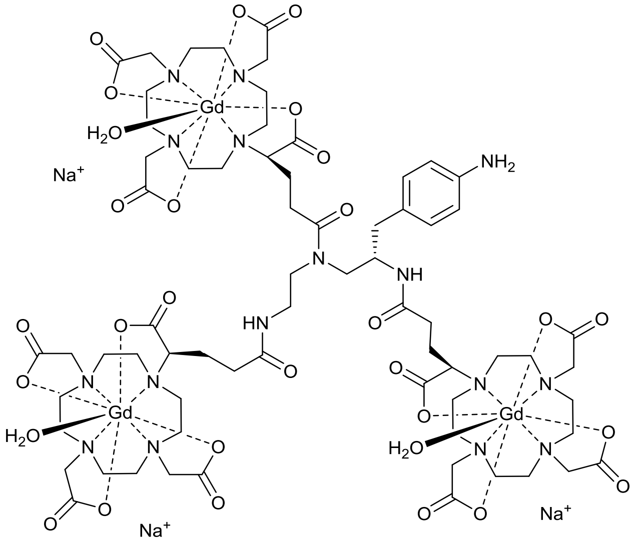

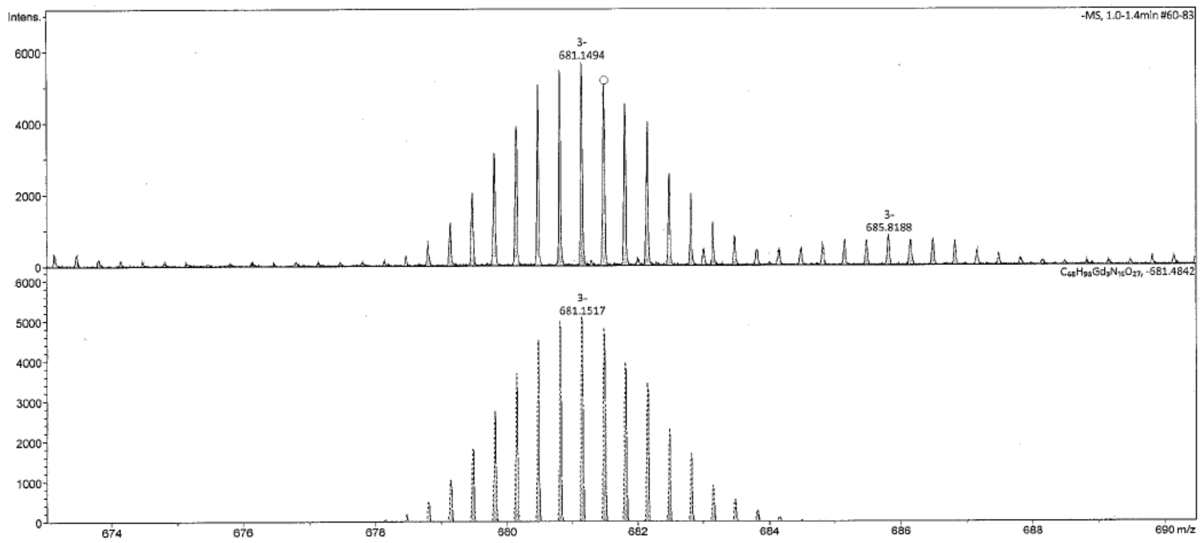

Top: measured HRMS (M^{3-})
Bottom: calculated HRMS (M^{3-})

[Gd-DOTA]$_3$-maleimide 13.

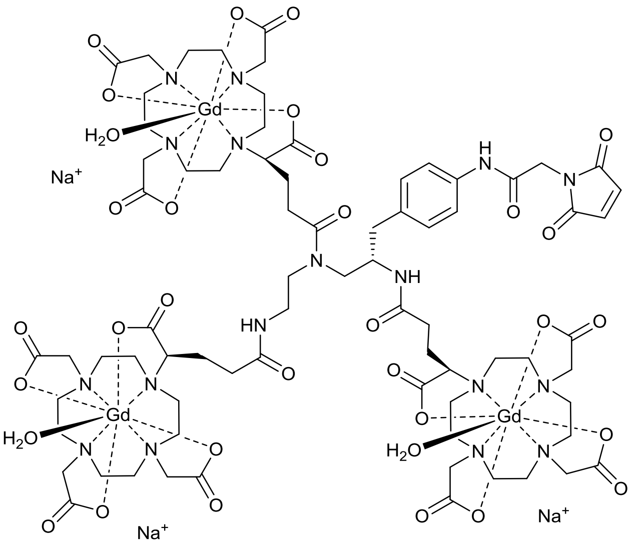

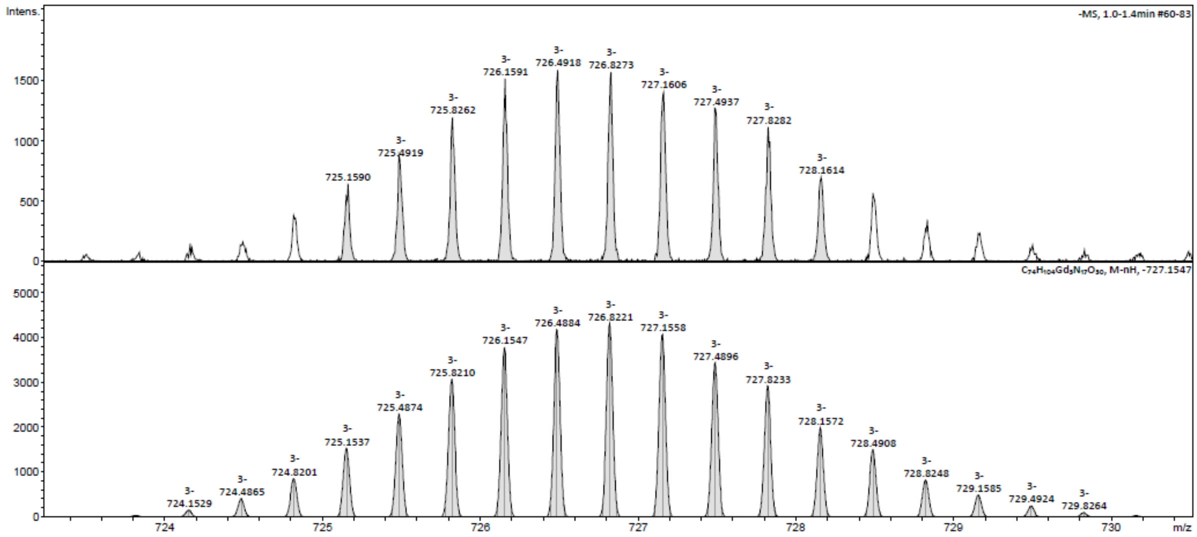

Top: measured HRMS (M^{3-})
Bottom: calculated HRMS (M^{3-})

[Gd-DOTA]$_3$-ITC 14.

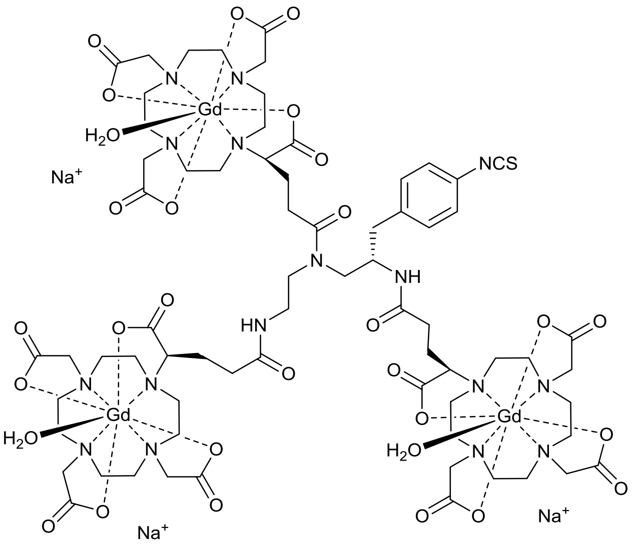

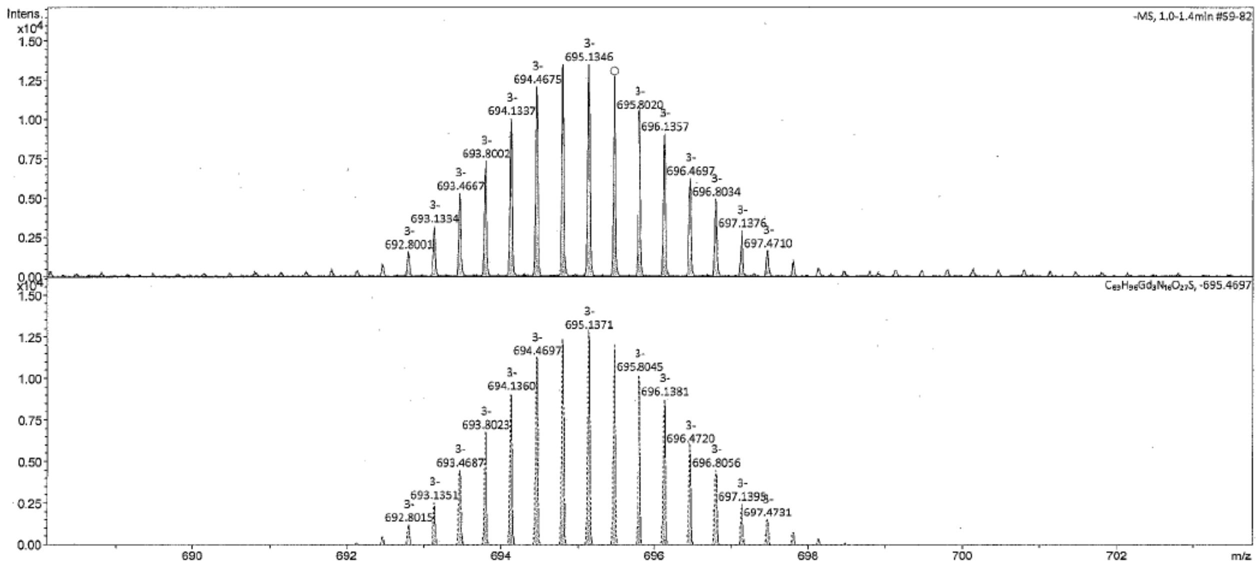

Top: measured HRMS (M^{3-})
Bottom: calculated HRMS (M^{3-})

[Gd-DOTA]$_3$-N$_3$ 15.

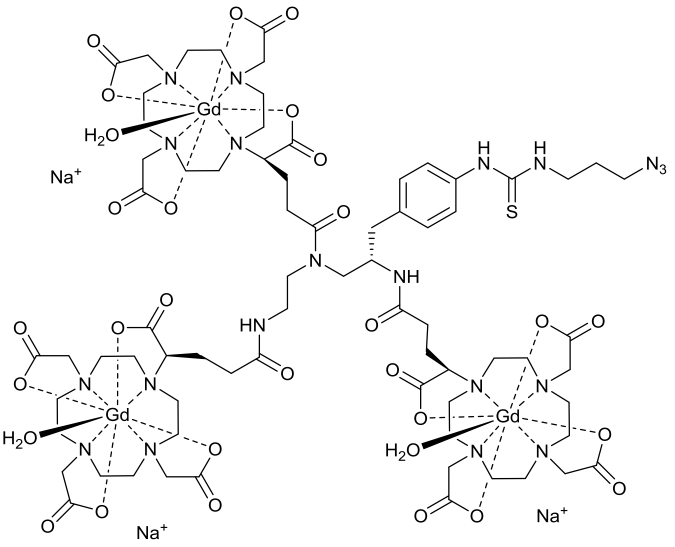

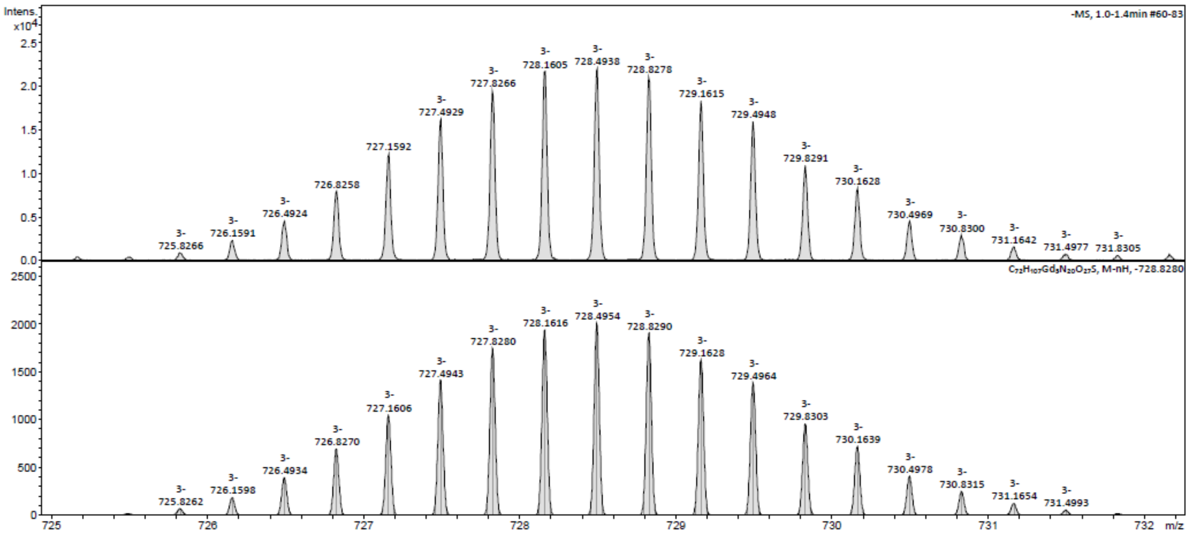

Top: measured HRMS (M^{3-})

Bottom: calculated HRMS (M^{3-})

4. Additional mass spectrometry data for labelled lysozyme

Unlabelled **lysozyme** (**A**): m/z

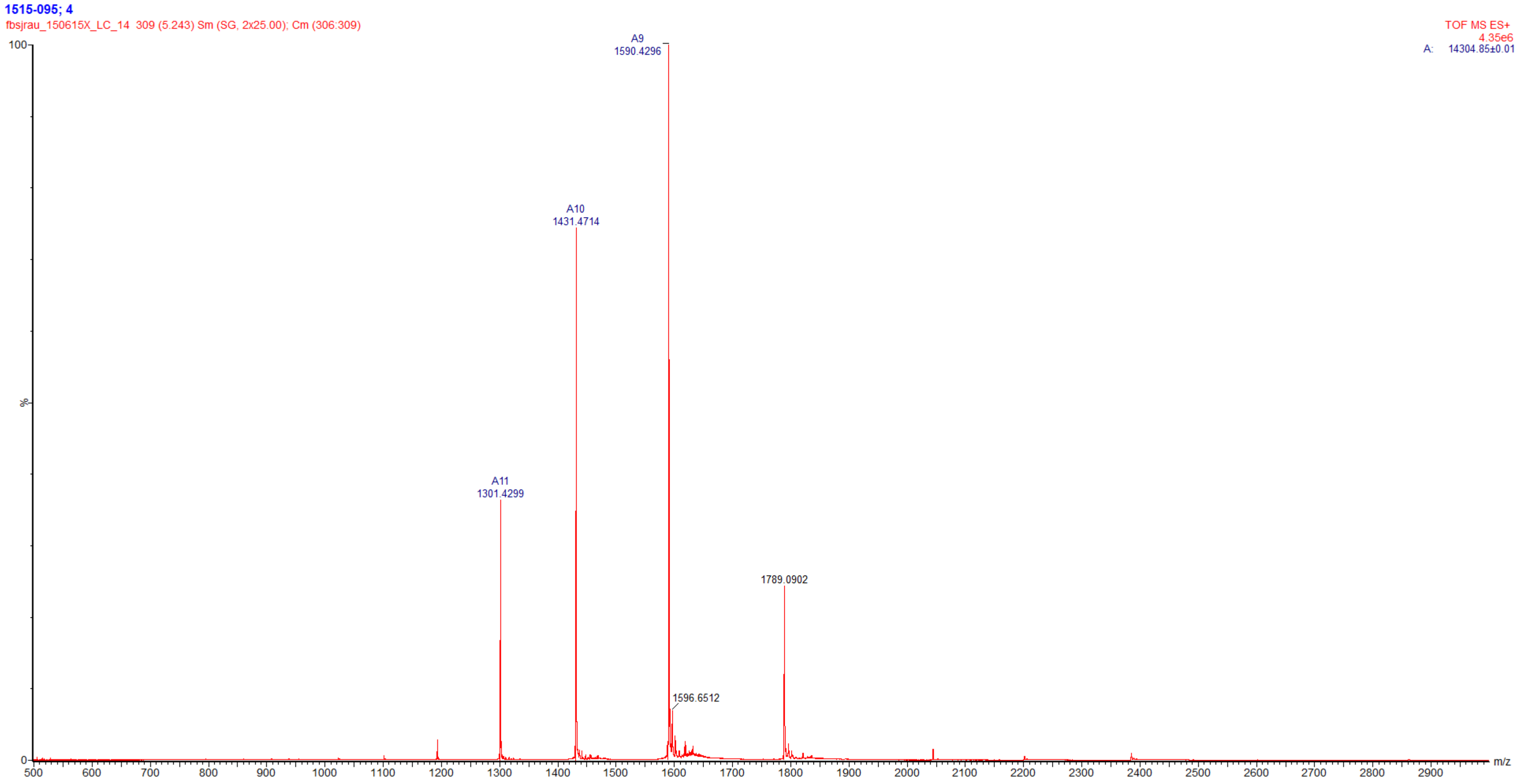

Unlabelled **lysozyme** (**A**): mass distribution

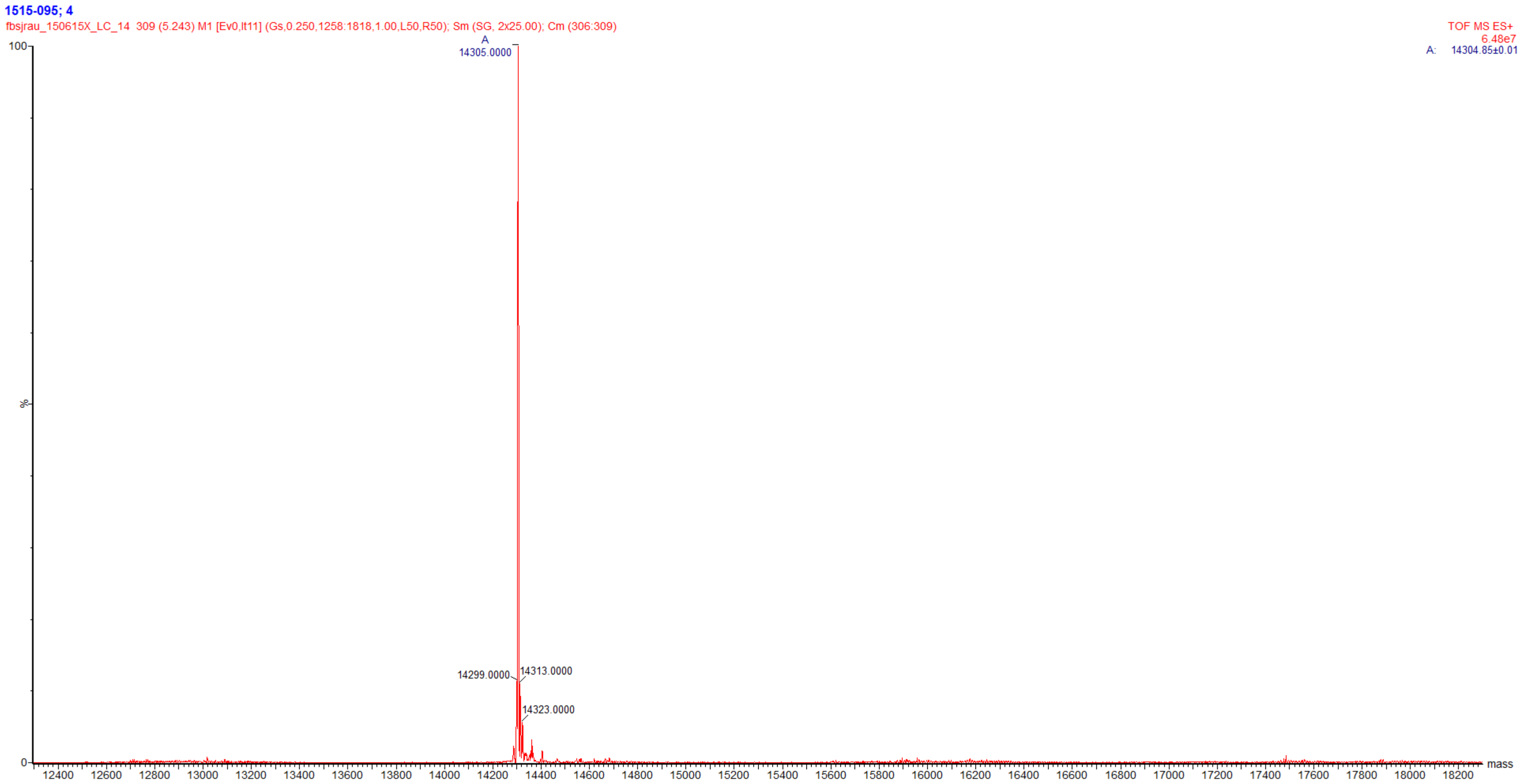

Reaction mixture containing **lysozyme-[Gd-DOTA]$_3$** (**A**), **lysozyme-([Gd-DOTA]$_3$)$_2$** (**B**) and **lysozyme-([Gd-DOTA]$_3$)$_3$** (not indicated): m/z

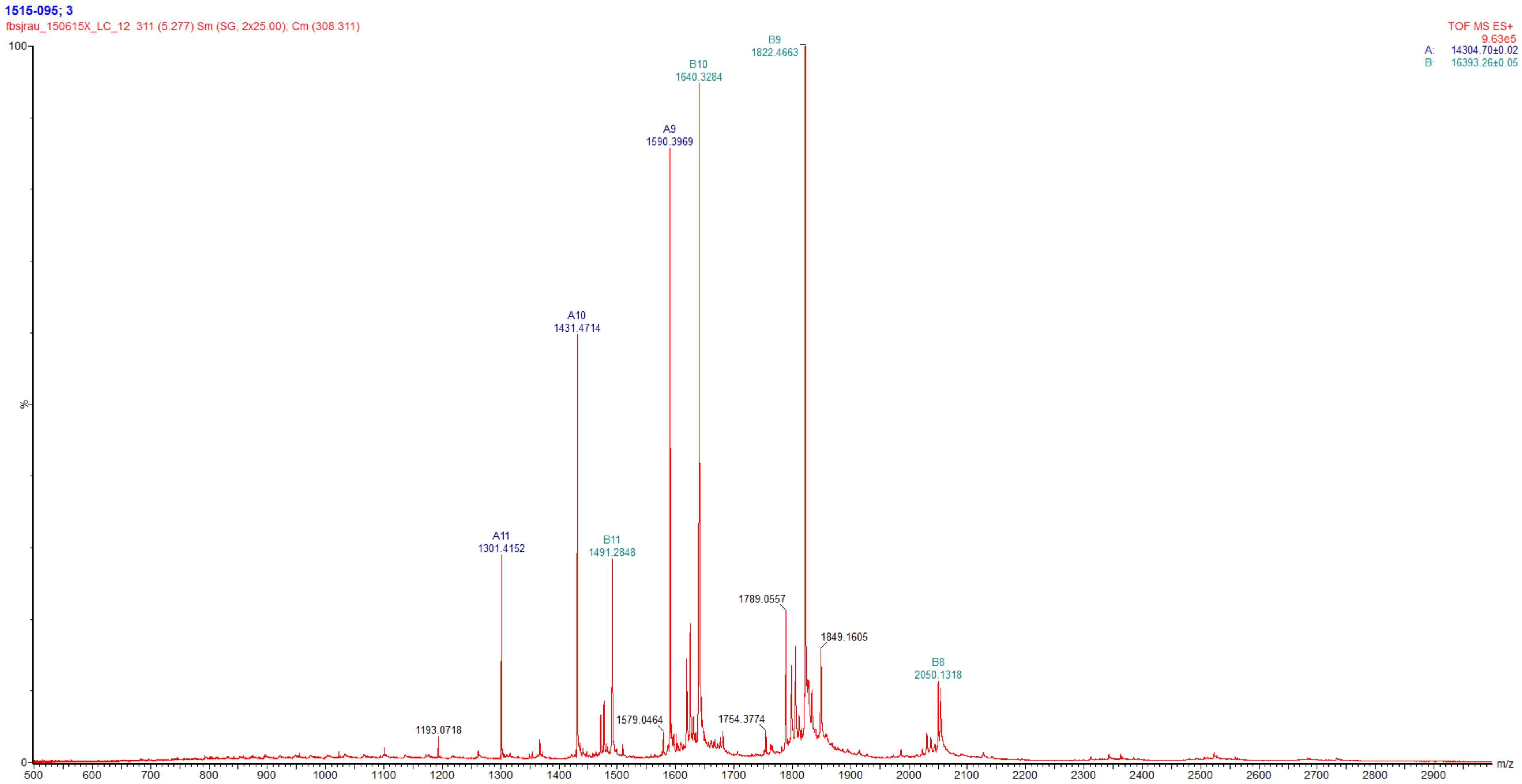

Reaction mixture containing **lysozyme-[Gd-DOTA]$_3$** (**A**), **lysozyme-([Gd-DOTA]$_3$)$_2$** (**B**) and **lysozyme-([Gd-DOTA]$_3$)$_3$** (**C**): mass distribution

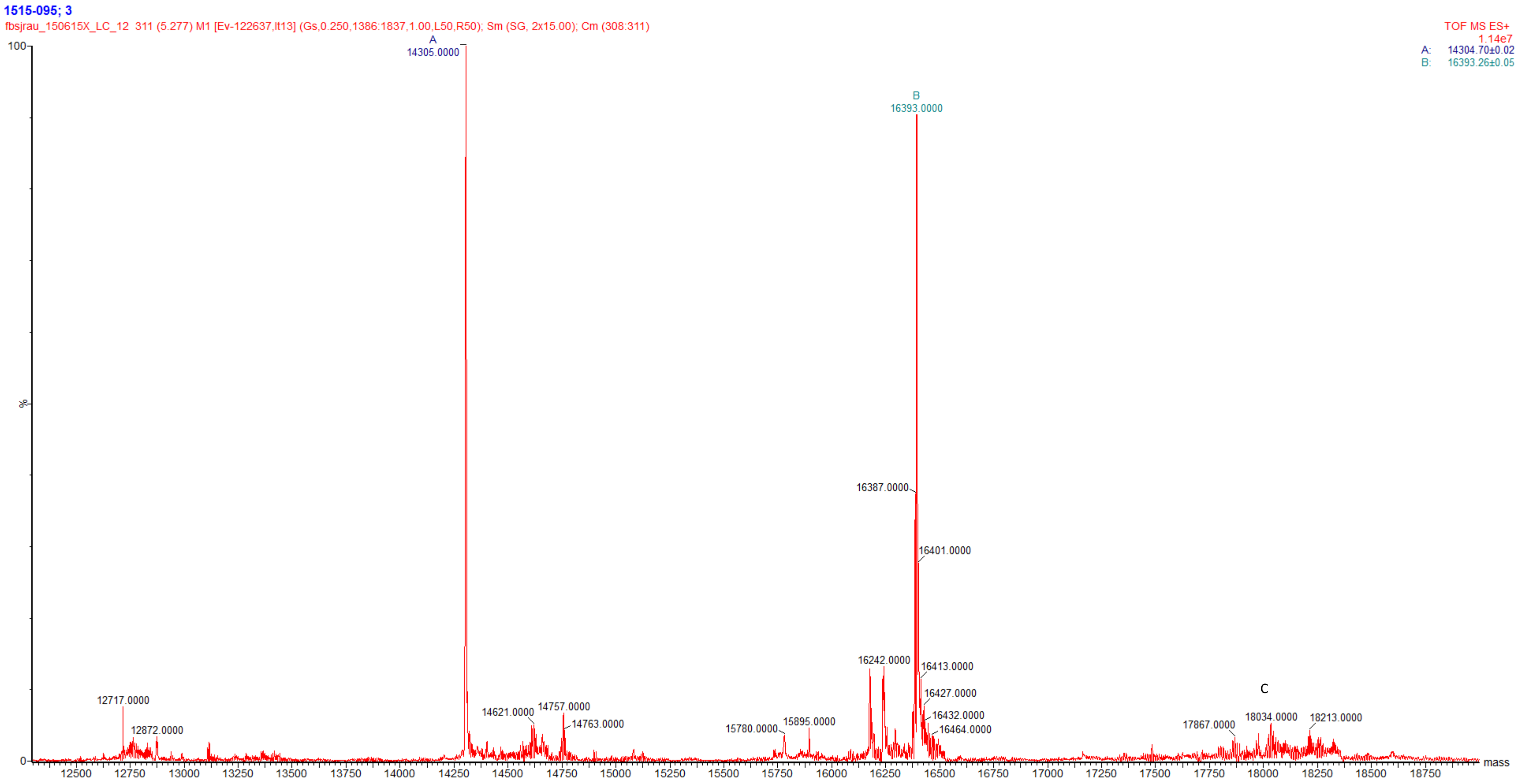

5. References

1. W.C. Still, M. Kahn and A. Mitra, *J. Org. Chem.*, 1978, **43**, 2923.
2. S.G. Levy, V. Jacques, K.L. Zhou, S. Kalogeropoulos, K. Schumacher, J.C. Amedio, J.E. Scherer, S.R. Witowski, R. Lombardy and K. Koppetsch, *Org. Process Res. Dev.*, 2009, **13**, 535.
3. B. Jagadish, G.L. Brickert-Albrecht, G.S. Nichol, E.A. Mash and N. Raghunand, *Tetrahedron Lett.*, 2011, **52**, 2058.
4. D.T. Corson and C.F. Meares, *Bioconjugate Chem.*, 2000, **11**, 292.
5. T.-P. Wang, N.C. Ko, Y.-C. Su, E.-C. Wang, S. Severance, C.-C. Hwang, Y.T. Shih, M.H. Wu and Y.-H. Chen, *Bioconjugate Chem.*, 2012, **23**, 2417.
6. Tiede C, Tang AA, Deacon SE, Mandal U, Nettleship JE, Owen RL, George SE, Harrison DJ, Owens RJ, Tomlinson DC, McPherson MJ, *Protein Eng. Des. Sel.*, 2014, **27**, 145.
